# Chemogenomic Analysis of G-Protein Coupled Receptors and Their Ligands Deciphers Locks and Keys Governing Diverse Aspects of Signalling

**DOI:** 10.1371/journal.pone.0016811

**Published:** 2011-02-04

**Authors:** Jörg D. Wichard, Antonius ter Laak, Gerd Krause, Nikolaus Heinrich, Ronald Kühne, Gunnar Kleinau

**Affiliations:** 1 Leibniz-Institut für Molekulare Pharmakologie, Berlin, Germany; 2 Institute of Experimental Pediatric Endocrinology, Charité Universitätsmedizin Berlin, Berlin, Germany; 3 Bayer-Schering Pharma, Berlin, Germany; University of Hong Kong, Hong Kong

## Abstract

Understanding the molecular mechanism of signalling in the important super-family of G-protein-coupled receptors (GPCRs) is causally related to questions of how and where these receptors can be activated or inhibited. In this context, it is of great interest to unravel the common molecular features of GPCRs as well as those related to an active or inactive state or to subtype specific G-protein coupling. In our underlying chemogenomics study, we analyse for the first time the statistical link between the properties of G-protein-coupled receptors and GPCR ligands. The technique of mutual information (MI) is able to reveal statistical inter-dependence between variations in amino acid residues on the one hand and variations in ligand molecular descriptors on the other. Although this MI analysis uses novel information that differs from the results of known site-directed mutagenesis studies or published GPCR crystal structures, the method is capable of identifying the well-known common ligand binding region of GPCRs between the upper part of the seven transmembrane helices and the second extracellular loop. The analysis shows amino acid positions that are sensitive to either stimulating (agonistic) or inhibitory (antagonistic) ligand effects or both. It appears that amino acid positions for antagonistic and agonistic effects are both concentrated around the extracellular region, but selective agonistic effects are cumulated between transmembrane helices (TMHs) 2, 3, and ECL2, while selective residues for antagonistic effects are located at the top of helices 5 and 6. Above all, the MI analysis provides detailed indications about amino acids located in the transmembrane region of these receptors that determine G-protein signalling pathway preferences.

## Introduction

G-protein-coupled receptors (GPCRs) constitute a large super-family of transmembrane receptors which convey extracellular signals into the intracellular region to effect sensory perception, chemotaxis, neurotransmission, cell communication and several other physiological events. The importance of GPCRs arises from their role as signal transmitters and regulators. In humans around 850 GPCRs are known [1] and several diseases are caused by GPCR malfunction [2]–[4]. They can be activated by a wide variety of endogenous stimuli such as amino acids, peptides, ions and (pher-) hormones [5]. GPCRs are subdivided into several families [6], whereby the largest family is the rhodopsin-like family A. Therefore, understanding these complex proteins and related signaling systems is of enormous importance, not least for drug discovery [7]–[11]. This is reflected by the fact that GPCRs are the largest target group for therapeutics [12] including up to 40% of currently marketed drugs [13].

Different structural parts of GPCRs are responsible for specific intra- as well as intermolecular functions during a sequential signal transduction process consisting of: i. receiving a stimulus, ii. transmission of the stimulus by inducing conformational changes of the receptor iii. intracellular presentation of determinants enabling activation of signal transducers such as G-proteins [14]. Most of the endogenous and synthetic ligands of family A GPCRs are thought to bind within the transmembrane domain close to the second extracellular loop 2 (ECL2) [11]. Based on a huge amount of experimental data a “global toggle switching” mechanism is assumed to take place during ligand induced activation, whereby a vertical see-saw movement of transmembrane helix (TMH) 6 occurs around a pivot [15], [16]. In consequence activation is characterized by a spatial re-arrangement of the TMHs and to the greatest extent between TMHs 5, 6, and 7 [17], [18]. This structural re-arrangement is supported by amino acids acting as “micro-switches” [19], [20]. In addition, contacts between ECL2 and the extracellular extensions of the helices have been proposed to participate as regulars during activation [21]–[23]. Different GPCR conformations are related to different signalling activity states [4], [19], [24] and several family A GPCR crystal structures were solved either in the inactive [18], [25]–[27] or the active conformation [28], [29].

On the intracellular side GPCRs interact with heterotrimeric guanine nucleotide-binding proteins (G-proteins), which play a crucial role in signal transduction towards second messenger cascades. G-protein subtypes are distinguished by their specific alpha-subunits. The main members are termed Gαs, Gαq and Gαi, whereby the induced effect on secondary messengers is considered (e.g. s-stimulation, or i-inhibition). GPCR mediated G-protein activation is characterized by structural shifts inside and between the G-protein subunits, followed by exchange of GDP for GTP in the α-subunit and separation of the Gα from the Gβγ-subunits. This opens up interfaces on the G-protein subunits to potential contact partners such as phospho-diesterase [30]. A detailed understanding of which structural features of GPCRs are related to selectivity of G-protein coupling and which factors or determinants are responsible for promiscuity in G-protein coupling of GPCRs is not yet available. The most prominent hypotheses addressing this topic are: 1) different conformational states of a receptor are selective for a certain G-protein subtype, since extracellular mutations and diverse ligands can cause different G-protein-subtype preferences for a single receptor [31]–[34]; 2) distinctive selective interaction patterns in terms of particular intracellular residues exist, which are responsible for G-protein subtype specific interactions [35]–[38]; 3) the G-protein preference is determined by the set of cell-specific G-protein subtype(s) [39]. None of these possibilities can be assigned as the main cause for G-protein preference for all GPCRs. Therefore, it is most likely that a combination of different factors define the G-protein portfolio of a certain GPCR.

The link between the variation in receptor properties and signal transduction specificities was studied using different theoretical methods. Earlier studies involving information theory measures for GPCR analysis focused on the receptor sequences and the interrelationship of the amino acid positions. Oliveira et al. used entropy-variability plots to detect functionally conserved residues [40]. Ye et al. proposed a two-entropy analysis to determine the functional positions in the transmembrane regions of GPCRs [41]. Fatakia et al. combined mutual information (MI) and concepts from graph theory to reveal correlated positions in the major GPCR classes [42].

The underlying study describes for the first time a chemogenomics approach on the activation mechanism and G-protein coupling of GPCRs. Chemogenomics is originally defined as a method that discovers active and/or selective ligands for biologically related targets in a systematic manner [43] and an extremely broad and significant example of chemogenomics is reported by Keiser et al. who quantitatively grouped and related hundreds of drug targets based on the chemical similarity of 65.000 ligands [44]. For GPCRs and other target families, Jabob and Vert proposed a more integrated chemogenomics method, as they apply their *Support Vector Machine* (SVM) machine learning algorithm to the joint chemical and the biological space, which is an advantage as it makes targets with few known ligands benefit from the data points of similar targets [45]. Recently, Van der Horst et al. presented a classification of GPCRs that is purely based on their ligands, complementing the classical sequence-based phylogenetic classifications of these receptors [46]. Such substructure-based and ligand-based phylogenetic classifications of GPCRs may help to unravel potential cross-reactivities of GPCR ligands.

The aim of this study is to identify features that link the sequence variation of family A GPCRs and their G-protein preference with the corresponding structural variation of agonistic and antagonistic ligands applying the concept of mutual information (MI). The novelty of our approach lies in the application of the MI concept to chemogenomics of GPCRs by including both the target sequence and ligand properties. In contrast to previous studies, we investigated the MI between GPCR residue variations in specific positions and the molecular properties of ligands. Furthermore, we also analyzed such receptor/ligand property correlations according to types of preferred G-protein activation. Strikingly, our analyses provide detailed indications about the effects of specific ligand properties and the determination of G-protein preferences in the transmembrane domain of GPCRs.

## Results

### Database, GPCR alignment, ligand description and mutual information

**Figure 1:**
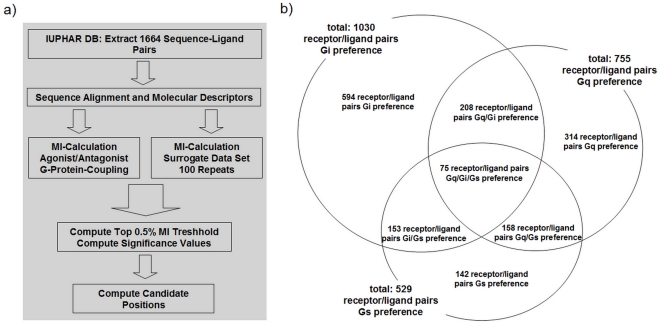
Extracted information from the IUPHAR database used for analyses. **a**) This workflow presents the methods we used to reveal the candidates with the highest mutual information between receptor positions and ligand features. **b**) We gathered the GPCR information from the *IUPHAR database* and extracted the 2D chemical structures from the *PubChem Structure Database*. In total 1664 receptor-ligand pairs were collected for analysis, whereby 100 different family A GPCRs from 30 sub-classes with known ligands were considered. Only full agonists, partial agonists and antagonists were explored. For the receptors their Gs, Gi and Gq coupling preferences were assigned based on the annotations in the *IUPHAR database*. Several receptors are known as to be promiscuous by their capability to activate two or three G-protein subtypes.

We gathered 100 family A GPCRs with known ligands and information regarding their preferred G-protein subtype from the standardized IUPHAR [47] database (figure 1a). These GPCRs cover over 30 receptor sub-families. Several of the extracted GPCRs couple only to a particular G-protein subtype, while most of them are able to undergo dual G-protein coupling to Gq/Gs, Gq/Gi or Gs/Gi pairs (figure 1b). Few receptors are known to activate all three G-protein subtypes. We assigned agonistic and antagonistic ligands to each receptor, whereby the individual receptor preference for G-protein coupling was also noted. More than 1660 receptor-ligand pairs containing 767 full agonists, 184 partial agonists and 713 antagonists were collected (figure 1b). In order to calculate the mutual information it is necessary to use appropriate descriptions of the sequence space and the ligand space. The representation of the sequence space is based on the multiple sequence alignment of the 100 analysed GPCRs using a profile hidden Markov model. The sequence alignment is provided under: http://fmp-berlin.info/research/structuralbiology/researchgroups/drug-design/downloads.html. To represent the ligand space we calculated a set of discrete and countable molecular descriptors (see section *Material and Methods*). The goal of mutual information consists in finding correlations between the variation of residues at a certain sequence position and the variation of ligand properties. Several data sets were built in order to calculate the MI for sequence-agonist, sequence-antagonists, sequence-Gi, sequence-Gs and sequence-Gq correlations. The data are shown tabulated in Table S1.

### Hot spot positions for antagonistic and agonistic effects on GPCRs

**Figure 2:**
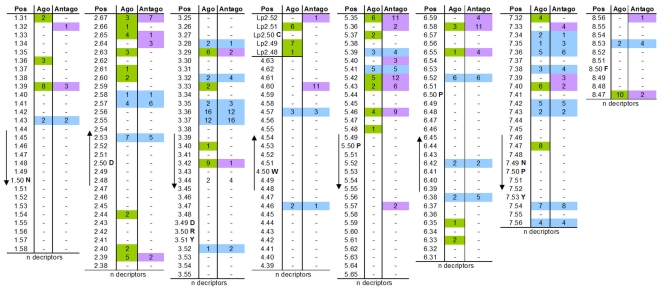
Identified positions for antagonistic and agonistic effects. This table summarizes the identified hot-spots of correlated GPCR residue positions and specific ligand properties by separating antagonistic and agonistic effects on signaling. Details can be found in Table S1. GPCR positions are given using the Ballesteros and Weinstein numbering [92]. For the second extracellular loop the highly conserved cysteine in ECL2, which in most family A GPCRs is linked to the cysteine in TMH3 (position 3.25), is numbered as Lp2.50. “Lp” indicates that this is a loop and number 2 that it is the second loop. For visualisation different colours are used according to the assigned functional effects: green - selective for agonists; lilac - selective for antagonist; blue - chemical descriptors of both agonists and antagonists are correlated and the number of descriptors is comparable. Highly conserved amino acids or motifs in each helix are provided in one letter code. n - number of descriptors that are correlated significantly.

**Figure 3:**
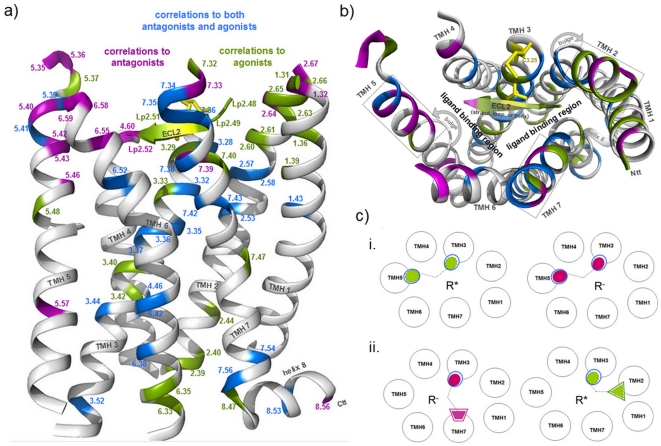
Identified positions for antagonistic and agonistic effects mapped to the rhodopsin crystal structure. **a**) GPCR positions correlated with chemical properties of ligands with either antagonistic or agonistic effects are mapped onto the inactive conformation of rhodopsin (pdb code 1U19). A colour code as in figure 2 enables the translation of functional information to the three-dimensional structure. The side-view with rendered backbone (white) highlights spatial localization and clustering of identified positions. **b**) The extracellular top view shows preferred ligand binding regions of family A GPCRs between the helices and the ECL2 close to the extracellular side. At the extracellular end especially of TMH2, 5 and 7 (boxes) several here identified amino acid side-chains are pointing towards the membrane. There involvement in ligand gating mechanisms or structural differences between the GPCRs can be speculated. **c**) Scheme of hypothetical scenarios for specific ligand-receptor interaction: i) both agonistic (green circular surface) or antagonistic effects (magenta circular surface) can be triggered at one receptor position (blue circle) dependent to the interrelated physico-chemical ligand properties. We assume that agonistic as well as antagonistic effects might be multiplied and simultaneously triggered at different parts of the receptor. ii) This also includes combinations between selective (triangle, trapeze) and non-selective contact points.

A relatively high value for the mutual information between receptor residue variations and ligand properties with respect to antagonistic or agonistic ligand induced effects, lead to the identification of common as well as selective GPCR positions. The MI-identified hot spot positions involved in ligand induced stabilization of the GPCR inactive state and ligand induced receptor activation are shown in figure 2. We have mapped this information onto the three-dimensional structure of rhodopsin for spatial assignment of sensitive positions (figure 3a). First of all, our analysis shows that the common family A GPCR ligand-binding region is highly sensitive to correlated residue-ligand properties (figure 3a,b). It is of special note that the side chains identified by our study are mostly oriented inwards towards the transmembrane helical bundle (figure 3a). Selective antagonistic positions are clustered around the extracellular region, especially between TMH5 and TMH6. Positions extracted for selective agonistic effects in the helices are mainly located between TMH1, TMH2 and TMH3 (e.g. 1.31, 1.36; 2.40, 2.44; 3.33) compared to selective antagonistic sensitive positions located mainly between TMH5 and TMH6 (e.g. 5.36, 5.40, 5.57).

Amino acids in the ECL2 (two residues before, and two behind the highly conserved cysteine which forms a disulphide bridge with cysteine 3.25 on TMH3) contribute significantly to ligand action, mostly with agonistic effects.

The amino-acid side chains at several positions point towards the membrane. These are located on the extracellular ends of TMHs 1, 2, 5 and 7. Our analysis also reveals amino acid positions which are most likely not involved in interactions with ligands, especially those that are located close to the intracellular side.

### Specific ligand properties can be assigned to antagonistic and/or agonistic effects

**Figure 4:**
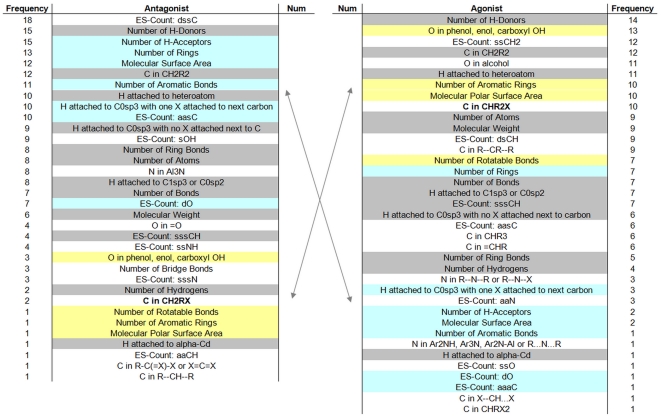
Frequency of descriptors for antagonists and agonists. The most significant ligand descriptors are sorted according to their frequency of occurrence in the results of our approach. Most of them are found for both types of ligands (coloured), agonistic and antagonistic, but few of them (white) are specifically observed only for one of each ligand-type. Interestingly, despite occurrence of descriptors for both ligand types to an equal amount (gray), few shared descriptors are found in high or low amount for agonists or antagonists (cyan, yellow), respectively.

The number of correlated ligand descriptors is in the range of 1-16 (figure 4). High numbers of correlated descriptors might be an indication for positions of priority in terms of ligand effect specificity. Several descriptors are found for both types of ligands (agonists and antagonists) but few are observable only for a particular type of ligand-effect. Interestingly, despite occurrence of shared descriptors to an equal amount, several of the descriptors assigned either to agonists or to antagonists do appear with different frequencies (high numbers versus low numbers). The fact that similar descriptors occur with different frequencies for the two effects could be an indication that these descriptors are related to ligand properties causing the agonistic or antagonistic response.

### Determination of G-protein preferences encoded in the transmembrane region of family A GPCRs

**Figure 5:**
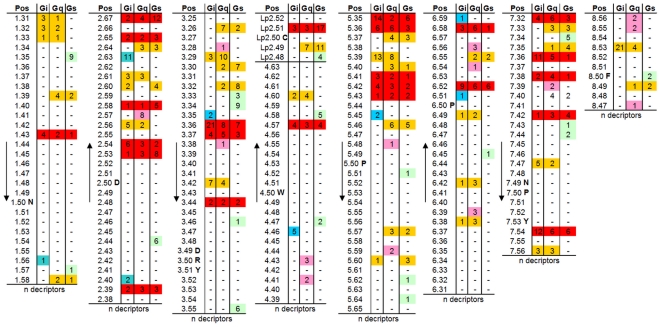
Receptor positions correlated with G-protein coupling preferences. In this figure the results of correlation-analysis between receptor residue variations and properties of ligands under separation of the receptor G-protein preference(s) are summarized. The highly conserved amino acids or motifs in each helix are provided in one letter code. The colour code highlights occurrence of single, double and also triple preferences of certain positions for G-protein coupling (Gs, Gi, Gq): red – correlations to all three G-protein subtypes; orange – preferences for any of the three possible Gq/Gs, Gi/Gs or Gi/Gq pairs; cyan – selective for Gi; magenta – selective for Gq; green – selective for Gs. n – number of descriptors are significantly contributing to the analysed effect (Table S1).

To investigate details of the general relationship between GPCR amino acid properties, ligands and the G-protein coupling preference we linked known G-protein (signaling pathway) preference to each particular receptor-ligand pair. We found three principal categories (figure 5):

1. Positions on all transmembrane helices are correlated to ligand properties and promiscuous G-preferences for Gi, Gq and Gs.

2. Selective amino acid positions can be found for Gs, Gq and Gi. Interestingly, this also includes the ECL2 and helix 8. The numbers of selective positions for the G-protein subtypes considered are: Gi-7, Gq-14, Gs-18.

3. Correlations of amino acid positions to pairs of Gi/Gq, Gs/Gq or Gi/Gs can be identified.

In cases 1. and 3. the number of correlated descriptor variables can vary to a high degree (e.g. 21 vs. 4) potentially indicating the priority of one out of two signaling pathways. This is the case for instance at position 3.36 with Gi n = 21, Gq n = 8, Gs n = 3, or position 8.53 with Gi n = 21, Gq n = 4.

## Discussion

Expansion of rhodopsin-like GPCRs started ∼500 million years ago [48]. Apart from a few exceptions such as the Glycoprotein hormone receptors (GPHRs) and Leucine-rich repeat containing G-protein coupled receptors (LGRs 4-8) [49], family A GPCRs are characterized by ligand binding close to or inside the transmembrane helices and the extracellular loops [11], [50], [51]. This is in contrast to other GPCR families and it has been proposed that this circumstance may be the basis for their evolutionary success, reflected by the highest number of members compared to the more structurally complex receptor proteins that have long ligand-binding N termini [9]. The advantages of such a ligand binding region might be the direct link to transmembrane signal transduction components (helices) and the general stability of the seven transmembrane helix structure which provides a scaffold for the evolution of new ligand binding partners. However, also for family B GPCRs few reports pointing to a potential allosteric ligand binding site between the transmembrane helices [52], [53].

An excellent analysis of this well-known classical transmembrane ligand binding region, supported by various mutagenesis studies, is given by Surgand and others for the entire collection of non-olfactive GPCRs [11]. These authors report a clear relationship between known ligand chemotypes (e.g. amines, carboxylic acids, phosphates, peptides, eicosanoids and lipids) and the cognate transmembrane cavities defined by 30 critical amino acid positions in the transmembrane helical region. Receptors for bulky ligands (e.g., phospholipids, prostanoids) appear to have a transmembrane cavity significantly larger than that for smaller compounds, and receptors for charged ligands (cationic amines, phosphates, mono and di-carboxylic acids) always present, among the 30 critical residues, one or more conserved amino acid exhibiting the opposite charge.

With exceptions such as rhodopsin which has a permanently bound inverse agonist, retinal, ligand binding is the initial event for signal transduction through the membrane via conformational changes in the helix arrangement [16], [19], [20], [27], [54]. Finally, heterotrimeric G-proteins are intracellularly activated which induces second messenger cascades [14], [30], [55]. Alternative signal transducers in addition to G-proteins are also known [56]. However, signal transduction across the membrane through GPCRs is governed by a complex set of interplaying components. A survey of their interrelationships is important for understanding the entire system. Several open but fundamental questions remain: Are there any distinct amino acid positions or spatial regions with a preference either for stimulation or inhibition of the signaling capability? Is the G-protein subtype preference of GPCRs dependent on specific ligand properties or subsequently on particular receptor-ligand contact positions?

The majority of the ligand-sensitive GPCR positions identified here are located and clustered (figure 3a,b) in the well-known transmembrane ligand binding region of family A GPCRs [57]. This includes residues in the ECL2, in agreement with a huge amount of published GPCR data [22], [58], [59]. Our study shows the ECL2 to be sensitive, especially to agonistic effects of ligands (figure 2). We found that several positions in the family A GPCRs investigated are correlated with both agonistic and antagonistic ligand effects. However, we also identified positions that are sensitive to only one effect. In this regard we would like to highlight positions with a high number of descriptors and a strong correlation to antagonistic signaling effects on the one hand: 2.64, 4.60, 5.36, 5.40, 6.58, 6.59, 7.33, 7.39, and a specific correlation to agonistic signaling effects on the other hand: 1.31, 1.36, 2.60, 2.63, 3.33, Lp2.49, Lp2.51, 7.32. Supporting this, polar interactions between amino acids in TMHs 1, 2, 3 and 7 are mandatory in family A GPCRs in order to constrain the inactive conformation [15], [16], [60], [61] which might also be true for family B GPCRs [62]. Water molecules are thought to participate in this bonding-network [63]–[65]. Therefore, activating effects induced by agonists must break these constraints to enable the shift towards the active state. In accordance with this assumption, the positions identified here that are correlated with specific agonistic effects are indeed located mainly in TMHs 1, 2, 3 and 7 (figure 3a,b). For the MC3R and MC4R ligand binding and activation sensitive residues specificly at TMHs 2, 3, 6 and TMH7 are reported [66]–[69]). A further prominent example is the A_2A_ adenosine receptor where Jaakola and co-workers have shown that specific hydrophobic and hydrophilic amino acid residues at TMH5 and TMH6 are important for action of both antagonistic and agonistic molecules [70], but they also concluded from previously published [71] and own data that in contrast to antagonistic molecules the ribose motif of the non-selective adenosine receptor agonist NECA binds exclusively to residues at TMH3 and TMH7. Studies at different GPCRs with specific ligands revealed sensitive residues for agonistic or antagonistic effects at each helix. At the 5-Hydroxytryptamine receptor (5HT2A) it was found, that agonistic action is induced by ligand interactions with residues at TMH5 and TMH6 [72], [73]. Furthermore, the crystal structure of the beta2-adrenergic receptor [74] complexed with the inverse agonist carazolol (pdb entry 2RH1) evidenced interactions (H-bonds) of this ligand to residues at TMH3, 5, and 7. This supports the conclusion (figure 3c) that specific combination of ligand/receptor contacts finally determine the effect on signaling.

Several similar ligand descriptors were found to be important for both agonists and antagonists and few of them occur with different frequencies (figure 4). We have also depicted ligand properties as contributing specifically to antagonistic or agonistic effects. Therefore, our analyses suggests that both agonistic and antagonistic effects can be triggered at the same receptor position depending on ligand properties (figure 3c i). Additionally combinations between non-selective and selective contacts between receptors and ligands are possible (figure 3c ii). Finally, the amino acid positions assigned to diverse effects induced by ligands reveal pharmacological patterns for agonists or antagonists.

It is of note that in contrast to the majority of inward pointing side-chains identified by our study, several hot-spot residues point out towards the membrane (figure 3a). They are located mainly at the extracellular ends of TMHs 2, 5 and 7 (figure 3b, boxes). One reason for the outward pointing side chains might be caused by structural differences between the GPCRs, because precisely these helices are characterized by a very diverse set of helix features [75] such as bulges and proline induced or supported kinks. These bulges and kinks have a strong effect on the helical twist and in consequence on the orientation of the side chains. In consequence, we assume that these regions and amino acids might be involved in ligand-gating mechanisms as shown in a previous study of Hildebrand and co-workers on opsin [76]. In conclusion, the mapping of our results for 100 different family A GPCRs onto one structural template, the rhodopsin structure, might not be generally representative for these particular regions. Equally, certain structural features in the extracellular regions of the transmembrane helices might be different for some of the family A GPCRs, despite the high structural overlap observable in the transmembrane region of the available GPCR crystal structures and our results may not be completely representative. Furthermore, our method has also identified residues outside the proposed general binding pocket region as correlated to specific ligand properties (figure 3a). They are mainly located close to or inside the intracellular region, which suggests their involvement in G-protein recognition and activation. Of special note are amino acids in helix 8. Their importance for GPCR signal transduction and G-protein coupling was pointed out in several studies on different GPCRs [37], [77]–[79]. In conclusion, the identified correlations between agonistic or antagonistic effects and positions outside the ligand-binding region are likely related to their importance for selective G-protein recognition or interaction with effectors.

**Figure 6:**
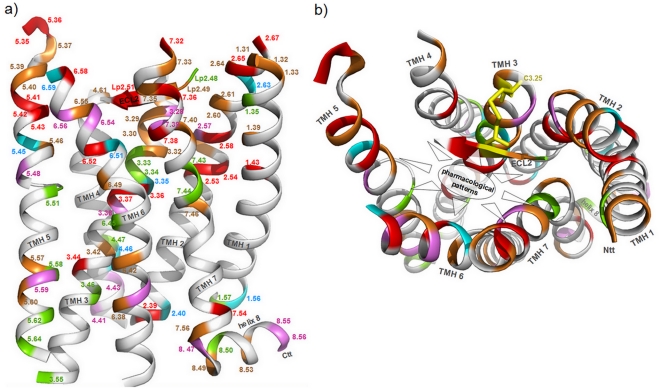
Receptor positions correlated with G-protein coupling preferences mapped to the rhodopsin crystal structure. **a**) The amino acid positions with highly correlated mutual information to specific ligand properties (antagonists and agonists) are mapped to the structure of rhodopsin (**b**, top-view), but in combination with the G-protein preference of a particular GPCR subtype indicated by specific colours according to figure 5.

Raymond [32] and Wess [39] have hypothesized from previous data that certain receptor amino acids inside the transmembrane region might be involved in the regulation of G-protein subtype preference. In 2000 it was shown experimentally for the β2-adrenergic receptor [33] that diverse ligands are indeed able to induce different signaling pathways at one receptor. By using FRET measurements on the α2-adrenergic receptor a correlation between diverse ligands and specific receptor conformations was recently shown [80]. Here we have found links between ligand effects and certain GPCR amino acid positions to be correlated with the activation of G-protein subtypes. Apart from the observation of amino acid positions at all transmembrane helices with correlations to effects on several G-protein subtypes (Gi, Gq, and Gs), we also revealed residues selective for either Gs, Gq or Gi (figures 5-6). We conclude that - comparable to the suggested scenario for agonistic or antagonistic effects - the ligand-specific effect on G-protein preference could be determined by the particular contact point(s) or the combination of receptor-ligand contacts [81], [82] (figure 3c). It might be that particular structural features on the intracellular side of the activated receptors are involved in regulation of G-protein subtype preference [24], [32], [37], [39], [83]. These structural features, including the intracellular surface-shape, are regulated by specific ligand-receptor contacts directing helical re-arrangements during activation. This regulation is constituted by induction or blocking of receptor movements. Subsequently, it would be feasible that Gs, Gi and Gq adjust slightly differently to the receptor conformation to allow optimal complementary intermolecular side-chain interactions in each case. The assumption and possibility of different active GPCR conformations, even if slight, is supported by several published data [24], [33], [34], [80], [84].

### Significance and limitations of the analyses

All findings, results and conclusions depend strongly on the quality of the database. The standardized IUPHAR database can be assumed to be one of the most complete collections of GPCR ligands. The IUPHAR database also contains GPCRs with promiscuous G-protein coupling capability (figure 2) but where the effect of dedicated ligands is not always evidenced by experimental studies on all types of G-protein coupling on that particular receptor. Therefore, it cannot be excluded that some receptor/ligand pairs assigned to a specific signaling pathway are potentially not linked to that pathway *in vivo*. As an example: for the LHCGR it was shown recently, that small allosteric ligands that bind to and activate this receptor do not induce both signaling pathways observed for the endogenous hormone-ligand [85].

The designated G-protein coupling (Gs, Gq, Gi) of the GPCRs are an indirect conclusion from measurements of signaling pathway intermediates like cAMP or calcium. The direct GPCR/G-protein interaction is hardly ever shown or proven. This is of relevance, because it has been discussed that the different subunits of a G-protein subtype may not be restricted to activating only one signaling pathway [30]. For example, for the lutropin receptor (LHCGR) it has been suggested that the beta-gamma subunit of Gi activates phospholipase C [86]. Therefore, exceptions might exist from the assumed direct one to one link between a signaling pathway and a specific G-protein subtype (subunit).

Highly conserved residues produce low values of mutual information and are therefore not included in the outcome of our analysis. This does of course not exclude their potential involvement in ligand-interaction or their participation in signal transduction.

In summary, we conducted this analysis with a maximum of data validation, but specific deviations from the general findings and conclusions are to be assumed given the statistical nature of our method and the restricted data set. A growing amount of reliable data will lead to deeper and more detailed insights.

Taken together, our results help with respect to set of limitations to understand GPCR signaling based on a distinct combination of interplaying parameters constituted by the chemical-physical properties of ligands and biophysical features of transmembrane GPCR residues. Our results can be condensed into three main hypotheses concerning regulation of signaling in family A GPCRs: 1. Residue positions in distinct receptor regions are specific for antagonistic or agonistic effects induced by ligands. 2. Important common ligand descriptors can be found for both types of ligands, but few are unique to either antagonists or agonists and might discriminate between the induced effects. 3. Preferences for activation of different G-protein subtypes are causally linked to specific residues in the transmembrane region of family A GPCRs. Our study, therefore, provides comprehensive information for understanding GPCR signaling and reveals new implications for the evolutionary derived intrinsic capacities of this protein super-family. We conducted this analysis with a maximum of data validation, but specific deviations from the general findings and conclusions are to be assumed given the statistical nature of our method and the restricted data set. A growing amount of reliable data will lead to deeper and more detailed insights.

## Materials and Methods

Our analysis follows the workflow that we show in figure 1a. After extraction of sequence-ligand pairs from the database, we align the sequences and calculate various molecular descriptors for the related ligands. For this data set we then calculate the mutual information between each alignment position and each molecular descriptor. For the significance test we repeat this calculation for 100 surrogate data sets. The top 0.5% of the mutual information values are selected as hot spot positions.

### The Database

Annotated compound libraries have emerged as a strong information basis for computational drug design and several vendors provide annotated libraries for different purposes [87]. There are several open-source databases available such as the *MOAD database* collected by Hu et al. [88], the *GLIDA database* by Okono et al. [89] or the *IUPHAR database* by Harmar et al. [47]. We gathered GPCR information from the *IUPHAR database* [47] and extracted the 2D chemical structures from the *PubChem Structure Database*. In total we collected 1664 receptor-ligand pairs covering 100 family A GPCRs (figure 1b). This collection represents the maximum amount of family A GPCRs with annotated ligands that was available from open source at the time of our study. Only full and partial agonists/antagonists were taken into account. Inverse agonists and allosteric modulators are not included in this study.

Additionally the known preferences for Gs, Gi and Gq activation which are given by the measured signalling pathway were assigned. Several receptors are known to be promiscuous for their ability to activate two or three G-protein subtypes (figure 1b).

### The GPCR sequence alignment

The Rhodopsin-like family A GPCRs share highly conserved residues in the seven helices [75]. Therefore the multiple sequence alignment of the 100 analysed GPCRs (available under: http://www.fmp-berlin.de/1129.html) is straightforward and was achieved for the transmembrane domain with a Profile Hidden Markov Model (HMM) as included in the HMMER software [90] and the PF00001 profile for the rhodopsin family taken from the *Pfam database* [91]. In addition, we adjusted the outcome of the HMM by hand in order to align the second extracellular loop (ECL2) around the cysteine residue involved in the highly conserved disulfide bridge between ECL2 and TMH3. Manual modifications were made to avoid gaps in the transmembrane helices. The extracted TMHs and conserved residues were numbered using the Ballesteros and Weinstein [92] numbering scheme to describe the common amino acid positions.

We also modified this numbering scheme by an extension to the extracellular (ECL) loop 2, whereby the highly conserved cysteine in ECL2, which in most family A GPCRs interacts with the cysteine in TMH3 (position 3.25), is numbered as Lp2.50. The “Lp” marks this as a loop and the “2” as the second loop. Estimation of these residues into analyses is supported by several previous studies on ligand interactions in GPCRs in the ECL2 [22].

### Molecular descriptors of analysed ligands

According to Todeschini and Consonni [93], a molecular descriptor is the ‘final result of a logical and mathematical procedure which transforms chemical information encoded within a symbolic representation of a molecule into a useful number or the result of some standardized experiment’.

In order to characterize the chemical space and the properties of the GPCR ligands we preferred discrete and countable descriptors such as element and molecular property counts, Ghose-Crippen AlogP [94] and electro-topological state counts [95], [96] which are easy to calculate using the two-dimensional structural formula. The only two continuous descriptors used were the molecular surface area and the molecular polar surface area. All molecular descriptors were calculated with the Pipeline Pilot software (http://www.scitegic.com).

### Mutual information

Mutual information is a basic concept in information theory and provides a general measure of interdependence between random variables.

If we consider a discrete random variable *X* with *n* possible values taken from the alphabet
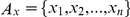
and the associated probability distribution 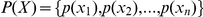 then the entropy *H(X)* is defined as
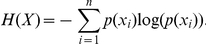



The joined entropy *H(X,Y)* of two random variables *X* and *Y* with the alphabet 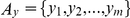 and the joined probability distribution
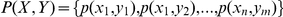
is defined as 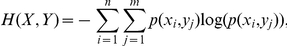
where 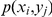 denotes the probability of the joined occurrence of 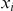 and 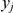. The mutual information *I*(*X*,*Y*) between two random variables *X* and *Y* is defined as
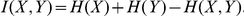



The mutual information is zero if *X* and *Y* are statistically independent. Since it makes no assumption about the type of relationship between *X* and *Y*, mutual information is sometimes considered to be an extension of the linear correlation coefficient [97].

The histogram approach is the most common way to estimate the mutual information of a finite sample set. It calculates the relative frequencies in the histogram bins as an estimate of the probability distribution of the random variables.

In general the total number and the width of the bins is crucial and has effects on the outcome of the estimation process [98]. Let us consider *K* simultaneous measurements of the two random variables *X* and *Y* with the alphabets 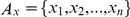 and 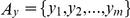 and let 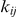 be the total number of measurements with 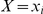 and 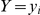. Then the probabilities 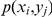 are approximated by the corresponding relative frequencies of the pairwise occurrence 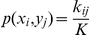 and accordingly for the single probabilities 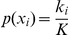 and 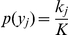.

The mutual information *I*(*X*,*Y*) of the two random variables *X* and *Y* turns into
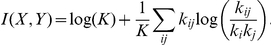



It is known that the estimation of information theoretical functions such as entropy and mutual information may be affected by systematic errors. Steuer et al. pointed out [98] that the systematic error could be fairly approximated by
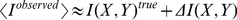
(1)with
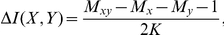
where 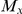,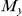 and 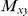 are the number of histogram bins with nonzero probability and 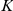
is the total number of data samples.

### Estimating the Mutual Information between Sequence Positions and Ligand Features

The random variable *X* is given by the distribution of the residues in a specific alignment position whereby the 20 natural amino acids define the canonical alphabet. The random variable *Y* is given by the distribution of a specific molecular descriptor and the alphabet is based on the discrete (countable) values. We selected the particular subsets for agonists, antagonists and the G-Protein coupling from the entire data set of 1664 ligand-receptor pairs and calculated the mutual information between each alignment position and each molecular descriptor. Only the top 0.5% of the mutual information values were selected and the results are reported in Table S1.

### Significance-Test

We formulate the null hypothesis that *X* and *Y* are independent. To test the null hypothesis we generate 100 different surrogate datasets 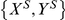 by creating random permutations of the original dataset 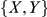. We calculate the corrected mutual information according to Equ. 1 for each surrogate realization of the null hypothesis and estimate the mean and the standard deviation 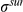.

The test statistic S is then given by
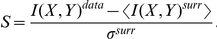



Under the assumption that the surrogate data follows a normal distribution we performed a one sided t-test at an alpha = 0.005 significance level which rejects the null hypothesis for S >2.63. The values for the test statistic are reported in Table S1.

We performed a *Lilliefors* test in order to check the assumption that the surrogate data sets follow a normal distribution. We test the default null hypothesis that the data set comes from a distribution in the normal family, against the alternative that it does not come from a normal distribution. The test rejects the null hypothesis at the 5% significance level in less than 10% of all cases and justifies the assumption that the surrogate data sets are normally distributed.

## Supporting Information

Table S1 (PDF)
