# Supplementary material for: Chemogenomic Analysis of G-Protein Coupled Receptors and Their Ligands Deciphers Locks and Keys Governing Diverse Aspects of Signalling
Source: PLoS One. 2011 Feb 4;6(2):e16811. doi: 10.1371/journal.pone.0016811 (PMC3033908; doi:10.1371/journal.pone.0016811)
Supplement: Table S1 — (PDF) [file pone.0016811.s001.pdf]

Position	Modus	Mutual Information	Test Statistic S	Molecular Descriptor
1.31	Agonist	0.3	9.91	ES-Count: sssCH
1.31	Agonist	0.3	11.08	ES-Count: aasC
1.31	Gi	0.24	11.16	Number of H-Acceptors
1.31	Gi	0.24	9.14	C in CH2R2
1.31	Gi	0.24	10.17	ES-Count: aasC
1.31	Gq	0.27	8.4	H attached to C0sp3 with one X attached to next carbon
1.32	Antagonist	0.2	6.1	Number of H-Donors
1.32	Gi	0.25	14.2	H attached to heteroatom
1.32	Gi	0.27	12.2	Number of Aromatic Rings
1.32	Gi	0.29	12.94	ES-Count: sssCH
1.32	Gq	0.27	8.61	Number of Positive Atoms
1.32	Gq	0.28	7.84	Molecular Surface Area
1.33	Gi	0.24	11.57	Molecular Weight
1.33	Gq	0.26	7.3	H attached to C0sp3 with no X attached next to C
1.35	Gs	0.28	18.99	ES-Count: sOH
1.35	Gs	0.29	17.78	Molecular Surface Area
1.35	Gs	0.29	6.25	O in phenol, enol, carboxyl OH
1.35	Gs	0.29	17.27	Number of H-Acceptors
1.35	Gs	0.29	9.52	Number of Atoms
1.35	Gs	0.3	10.49	N in Al2NH
1.35	Gs	0.3	11.69	Number of Rings
1.35	Gs	0.32	9.4	H attached to alpha-Cd
1.35	Gs	0.33	12.98	ES-Count: sssCH
1.36	Agonist	0.29	15.84	Number of Bonds
1.36	Agonist	0.31	22.56	Number of Atoms
1.36	Agonist	0.32	18.09	C in CH2R2
1.39	Agonist	0.29	13.6	C in =CHR
1.39	Agonist	0.31	18.58	Number of Hydrogens
1.39	Agonist	0.31	26.22	Number of Rotatable Bonds
1.39	Agonist	0.31	26.2	O in alcohol
1.39	Agonist	0.33	18.48	Number of Rings
1.39	Agonist	0.36	22.87	C in CHR2X
1.39	Agonist	0.38	19.77	C in CH2R2
1.39	Agonist	0.39	19.95	C in R--CR--R
1.39	Antagonist	0.2	7.75	ES-Count: dssC
1.39	Antagonist	0.2	9.98	N in Al3N
1.39	Antagonist	0.21	12.76	ES-Count: aasC
1.39	Gq	0.28	22.1	C in CHR3
1.39	Gq	0.34	16.69	C in CH2R2
1.39	Gq	0.34	14.58	C in =CHR
1.39	Gq	0.37	19.73	ES-Count: sssCH
1.39	Gs	0.29	11.58	N in Al3N
1.39	Gs	0.29	7.8	Number of Bonds
1.43	Agonist	0.31	16.8	C in CH2R2
1.43	Agonist	0.32	21.64	Number of Rings
1.43	Antagonist	0.21	8.68	ES-Count: dssC
1.43	Antagonist	0.22	7.92	N in Al3N
1.43	Gi	0.24	27.48	Number of H-Acceptors
1.43	Gi	0.25	15.36	Molecular Polar Surface Area
1.43	Gi	0.26	15.52	ES-Count: ssCH2
1.43	Gi	0.28	22.14	O in phenol, enol, carboxyl OH
1.43	Gq	0.27	11.9	Molecular Weight

Position	Modus	Mutual Information	Test Statistic S	Molecular Descriptor
1.43	Gq	0.28	13.1	Molecular Surface Area
1.43	Gs	0.3	28.01	Number of Rings
1.56	Gi	0.23	34.14	Number of Aromatic Bonds
1.57	Gs	0.28	18.08	C in R--CH--R
1.58	Gq	0.26	8.04	Molecular Weight
1.58	Gq	0.28	9.83	Number of Rings
1.58	Gs	0.3	9.82	C in R--CX--X
2.39	Agonist	0.29	27.35	O in alcohol
2.39	Agonist	0.29	22.56	Number of Rings
2.39	Agonist	0.33	22.33	O in phenol, enol, carboxyl OH
2.39	Agonist	0.34	17.9	ES-Count: ssCH2
2.39	Agonist	0.35	21.18	C in CHR3
2.39	Antagonist	0.21	6.48	ES-Count: dssC
2.39	Antagonist	0.22	8.88	Number of H-Donors
2.39	Gi	0.25	18.94	H attached to C1sp3 or C0sp2
2.39	Gi	0.27	16.18	ES-Count: sssCH
2.39	Gq	0.28	16.62	O in phenol, enol, carboxyl OH
2.39	Gq	0.29	12.03	Number of Bonds
2.39	Gq	0.31	11.07	Number of Atoms
2.39	Gs	0.28	16.38	Molecular Polar Surface Area
2.39	Gs	0.28	10.95	H attached to alpha-Cd
2.39	Gs	0.3	17.99	Number of Bonds
2.40	Agonist	0.29	15.07	ES-Count: sssCH
2.40	Agonist	0.31	26.35	Number of Rings
2.40	Gi	0.24	10.7	ES-Count: sssCH
2.40	Gi	0.25	14.13	O in =O
2.44	Agonist	0.3	16.66	Number of Rotatable Bonds
2.44	Agonist	0.3	14.94	O in phenol, enol, carboxyl OH
2.44	Gs	0.28	8.72	Molecular Surface Area
2.44	Gs	0.3	10.04	Number of H-Acceptors
2.44	Gs	0.31	9.04	Number of Atoms
2.44	Gs	0.32	12.25	Molecular Weight
2.44	Gs	0.32	12.42	Number of Bonds
2.44	Gs	0.32	14.52	H attached to alpha-Cd
2.53	Agonist	0.29	20.3	O in alcohol
2.53	Agonist	0.3	16.82	C in CHR3
2.53	Agonist	0.31	22.04	ES-Count: sssCH
2.53	Agonist	0.32	17.61	Number of H-Acceptors
2.53	Agonist	0.32	21.25	C in R--CR--R
2.53	Agonist	0.34	31.83	Number of Atoms
2.53	Agonist	0.35	30.4	Number of Bonds
2.53	Antagonist	0.21	16.63	H attached to C0sp3 with one X attached to next carbon
2.53	Antagonist	0.21	10.93	C in CH2R2
2.53	Antagonist	0.22	9.34	Molecular Surface Area
2.53	Antagonist	0.22	12.07	ES-Count: dssC
2.53	Antagonist	0.23	9.5	ES-Count: sssN
2.53	Gi	0.23	20.31	H attached to alpha-Cd
2.53	Gq	0.27	20.21	ES-Count: aasC
2.53	Gq	0.29	14.63	Number of Hydrogens
2.53	Gq	0.3	17.89	Molecular Surface Area
2.53	Gs	0.28	18.9	Number of Rings
2.53	Gs	0.29	11.56	N in Al2NH
2.53	Gs	0.29	23.13	O in phenol, enol, carboxyl OH

Position	Modus	Mutual Information	Test Statistic S	Molecular Descriptor
2.53	Gs	0.29	20.05	C in CHR3
2.53	Gs	0.29	13.53	ES-Count: aasC
2.53	Gs	0.3	14.4	Molecular Surface Area
2.53	Gs	0.32	10.78	Number of Atoms
2.53	Gs	0.34	17.62	Number of H-Acceptors
2.54	Gi	0.24	15.84	ES-Count: sOH
2.54	Gi	0.24	17.19	H attached to C0sp3 with no X attached next to C
2.54	Gi	0.25	17.99	H attached to heteroatom
2.54	Gi	0.25	15.27	N in RCO-N< or >N-X=X
2.54	Gi	0.26	14.42	Number of Ring Bonds
2.54	Gi	0.26	18.95	Molecular Polar Surface Area
2.54	Gq	0.27	14.98	Number of Hydrogens
2.54	Gq	0.28	10.77	ES-Count: dssC
2.54	Gq	0.31	11.33	ES-Count: sssCH
2.54	Gs	0.29	14.51	Number of Rings
2.54	Gs	0.31	10.99	C in CHR3
2.56	Gi	0.23	14.67	ES-Count: ssNH
2.56	Gi	0.23	18.97	Number of H-Acceptors
2.56	Gi	0.25	19.84	Number of Rotatable Bonds
2.56	Gi	0.25	18.37	H attached to heteroatom
2.56	Gi	0.26	15.22	ES-Count: ssCH2
2.56	Gq	0.28	13.26	ES-Count: ssNH
2.56	Gq	0.3	10.81	Number of Rotatable Bonds
2.57	Agonist	0.31	24.75	Molecular Weight
2.57	Agonist	0.31	21.14	C in CHR2X
2.57	Agonist	0.38	19.98	C in CH2R2
2.57	Agonist	0.41	22.67	O in phenol, enol, carboxyl OH
2.57	Antagonist	0.21	8.74	O in phenol, enol, carboxyl OH
2.57	Antagonist	0.21	7.46	ES-Count: dssC
2.57	Antagonist	0.21	17.33	Number of Aromatic Bonds
2.57	Antagonist	0.23	9.65	H attached to heteroatom
2.57	Antagonist	0.24	9.71	Number of Rings
2.57	Antagonist	0.25	14.73	Number of Bridge Bonds
2.57	Gq	0.26	18.4	ES-Count: sssCH
2.57	Gq	0.26	17.84	O in =O
2.57	Gq	0.27	22.06	Molecular Weight
2.57	Gq	0.29	14.68	Molecular Surface Area
2.57	Gq	0.3	19.74	Number of Atoms
2.57	Gq	0.36	19.56	Number of Bonds
2.57	Gq	0.37	16.33	ES-Count: dO
2.57	Gq	0.38	14.75	C in CH2R2
2.58	Agonist	0.31	25.37	C in CHR3
2.58	Antagonist	0.23	22.56	Number of Rings
2.58	Gi	0.23	27.42	H attached to heteroatom
2.58	Gq	0.29	23.7	Molecular Weight
2.58	Gs	0.28	14.88	Number of H-Acceptors
2.58	Gs	0.28	9.14	ES-Count: sssCH
2.58	Gs	0.29	19.62	ES-Count: sssN
2.58	Gs	0.29	21.6	O in phenol, enol, carboxyl OH
2.58	Gs	0.29	18.68	H attached to alpha-Cd
2.60	Agonist	0.3	16.96	Molecular Polar Surface Area
2.60	Agonist	0.32	17.73	H attached to C1sp3 or C0sp2
2.60	Gi	0.23	12.75	H attached to alpha-Cd

Position	Modus	Mutual Information	Test Statistic S	Molecular Descriptor
2.60	Gi	0.23	13.59	Number of Bridge Bonds
2.60	Gs	0.29	10.11	Number of Rings
2.60	Gs	0.3	11.08	Number of Ring Assemblies
2.60	Gs	0.3	10.72	Number of Atoms
2.60	Gs	0.31	11.31	Molecular Weight
2.61	Agonist	0.29	18.73	H attached to C0sp3 with one X attached to next carbon
2.61	Gi	0.23	13.03	Number of H-Acceptors
2.61	Gi	0.27	18.11	Molecular Polar Surface Area
2.61	Gi	0.29	17.77	ES-Count: dssC
2.61	Gq	0.26	12.5	Molecular Weight
2.61	Gq	0.29	11.58	Number of Atoms
2.61	Gq	0.3	17.78	Molecular Surface Area
2.63	Agonist	0.28	19.1	Molecular Polar Surface Area
2.63	Agonist	0.29	10.91	ES-Count: ssCH2
2.63	Agonist	0.31	12.28	Number of Bonds
2.63	Gi	0.23	10.88	Molecular Weight
2.63	Gi	0.23	12.26	C in CHR2X
2.63	Gi	0.23	19.68	Number of Rotatable Bonds
2.63	Gi	0.24	11.05	H attached to alpha-Cd
2.63	Gi	0.24	10.87	Number of Hydrogens
2.63	Gi	0.24	11.83	Molecular Surface Area
2.63	Gi	0.25	11.67	ES-Count: dO
2.63	Gi	0.26	13.78	Number of H-Acceptors
2.63	Gi	0.26	12.69	O in =O
2.63	Gi	0.27	10.78	C in CH2R2
2.63	Gi	0.29	11.9	Molecular Polar Surface Area
2.64	Antagonist	0.21	4.22	O in =O
2.64	Antagonist	0.22	7.19	Number of H-Acceptors
2.64	Antagonist	0.22	6.39	N in Al3N
2.64	Gq	0.3	10.11	Number of Rings
2.64	Gq	0.32	9.61	ES-Count: aaaC
2.64	Gq	0.33	10.52	Molecular Surface Area
2.64	Gs	0.29	9.62	H attached to heteroatom
2.64	Gs	0.29	9.57	Molecular Polar Surface Area
2.64	Gs	0.29	10.9	N in R--N--R or R--N--X
2.65	Agonist	0.3	23.32	Molecular Polar Surface Area
2.65	Agonist	0.32	18.19	Number of H-Donors
2.65	Agonist	0.33	15.83	O in alcohol
2.65	Agonist	0.37	23.03	ES-Count: aasC
2.65	Antagonist	0.2	5.13	Number of Rings
2.65	Gi	0.23	15.28	Number of Aromatic Rings
2.65	Gi	0.24	13.85	Molecular Weight
2.65	Gq	0.28	9.46	Molecular Surface Area
2.65	Gq	0.29	10.15	Number of Atoms
2.65	Gs	0.28	9.93	C in CHR3
2.65	Gs	0.32	10.57	Molecular Polar Surface Area
2.65	Gs	0.34	10.02	H attached to C0sp3 with one X attached to next carbon
2.66	Agonist	0.29	21.33	H attached to C1sp3 or C0sp2
2.67	Agonist	0.28	10.06	Number of Aromatic Rings
2.67	Agonist	0.3	10.59	C in CHRX2
2.67	Agonist	0.35	21.55	ES-Count: aaN
2.67	Antagonist	0.2	6.45	Molecular Surface Area

Position	Modus	Mutual Information	Test Statistic S	Molecular Descriptor
2.67	Antagonist	0.2	6.14	Number of Aromatic Bonds
2.67	Antagonist	0.2	9.34	Number of H-Donors
2.67	Antagonist	0.21	6.39	ES-Count: dssC
2.67	Antagonist	0.21	4.18	ES-Count: aasC
2.67	Antagonist	0.22	5.32	C in CH2R2
2.67	Antagonist	0.23	3.67	H attached to C0sp3 with one X attached to next carbon
2.67	Gi	0.23	8.91	C in CHR2X
2.67	Gi	0.25	9.88	Molecular Surface Area
2.67	Gq	0.28	8.49	ES-Count: sssCH
2.67	Gq	0.29	12.24	ES-Count: aaN
2.67	Gq	0.29	17.49	Number of Rings
2.67	Gq	0.31	8.9	C in =CHR
2.67	Gs	0.29	7.15	Number of H-Acceptors
2.67	Gs	0.29	6.97	C in CH2R2
2.67	Gs	0.31	8.23	H attached to C1sp3 or C0sp2
2.67	Gs	0.31	15.31	C in =CHR
2.67	Gs	0.32	13.99	Number of Rings
2.67	Gs	0.33	5.47	Molecular Polar Surface Area
2.67	Gs	0.33	6.46	ES-Count: dsCH
2.67	Gs	0.34	10.18	ES-Count: aaN
2.67	Gs	0.34	9.31	N in R--N--R or R--N--X
2.67	Gs	0.35	8.58	Number of Aromatic Rings
2.67	Gs	0.38	8.52	C in CH2RX
2.67	Gs	0.4	10.24	ES-Count: aasC
3.26	Gq	0.26	14.09	Molecular Weight
3.26	Gq	0.28	18.3	Number of Ring Bonds
3.26	Gq	0.28	14.62	Molecular Surface Area
3.26	Gq	0.29	21.17	O in phenol, enol, carboxyl OH
3.26	Gq	0.3	11.68	C in CH2R2
3.26	Gq	0.3	11.24	O in =O
3.26	Gq	0.34	15.61	ES-Count: dO
3.26	Gs	0.28	10.11	Number of Atoms
3.26	Gs	0.3	11.74	ES-Count: aasC
3.28	Agonist	0.3	23.31	O in alcohol
3.28	Agonist	0.3	13.47	Molecular Surface Area
3.28	Antagonist	0.24	11.38	Number of Aromatic Bonds
3.28	Gq	0.29	23.34	ES-Count: aaN
3.29	Agonist	0.3	15.28	ES-Count: ssCH2
3.29	Agonist	0.31	19.65	H attached to C0sp3 with no X attached next to C
3.29	Agonist	0.32	19.84	Number of Bonds
3.29	Agonist	0.33	16.88	H attached to C0sp3 with one X attached to next carbon
3.29	Agonist	0.33	16.92	Molecular Weight
3.29	Agonist	0.35	25.09	Number of Atoms
3.29	Antagonist	0.22	11.75	H attached to C1sp3 or C0sp2
3.29	Antagonist	0.23	13.89	H attached to C0sp3 with no X attached next to C
3.29	Gi	0.23	18.76	Number of Hydrogens
3.29	Gi	0.24	18.22	ES-Count: ssCH2
3.29	Gi	0.26	23.27	Molecular Surface Area
3.29	Gq	0.26	20.74	Molecular Weight
3.29	Gq	0.26	20.08	Number of Bonds

Position	Modus	Mutual Information	Test Statistic S	Molecular Descriptor
3.29	Gq	0.27	18.72	ES-Count: dO
3.29	Gq	0.27	18.27	H attached to alpha-Cd
3.29	Gq	0.27	14.49	N in RCO-N< or >N-X=X
3.29	Gq	0.29	13.51	ES-Count: dssC
3.29	Gq	0.29	18.09	H attached to C0sp3 with no X attached next to C
3.29	Gq	0.29	14.9	O in =O
3.29	Gq	0.29	18.09	Number of Hydrogens
3.29	Gq	0.31	14.24	C in CH2R2
3.30	Gq	0.26	12.35	ES-Count: dsCH
3.30	Gq	0.27	10.34	O in phenol, enol, carboxyl OH
3.30	Gs	0.28	11.39	C in CHR3
3.30	Gs	0.3	11.08	N in Al3N
3.30	Gs	0.32	25.97	O in phenol, enol, carboxyl OH
3.30	Gs	0.32	18.1	ES-Count: sssN
3.30	Gs	0.33	16.34	ES-Count: sssCH
3.30	Gs	0.34	11.47	N in R--N--R or R--N--X
3.30	Gs	0.34	14.24	Number of H-Acceptors
3.32	Agonist	0.29	20.25	N in R--N--R or R--N--X
3.32	Agonist	0.31	29.42	ES-Count: dsCH
3.32	Antagonist	0.2	21.42	C in R--CH--R
3.32	Antagonist	0.2	10.75	C in R-C(=X)-X or X=C=X
3.32	Antagonist	0.22	24.01	ES-Count: aaCH
3.32	Antagonist	0.24	18.43	Number of Aromatic Bonds
3.32	Gq	0.26	18.45	ES-Count: ssCH2
3.32	Gq	0.26	20.91	C in CH2R2
3.32	Gs	0.29	9.87	Number of Bonds
3.32	Gs	0.29	10.44	Number of Aromatic Bonds
3.32	Gs	0.29	13.32	H attached to alpha-Cd
3.32	Gs	0.29	16.47	Number of Hydrogens
3.32	Gs	0.29	10.13	C in =CHR
3.32	Gs	0.3	15.26	ES-Count: dsCH
3.32	Gs	0.31	13.62	O in alcohol
3.32	Gs	0.34	16.16	C in CHR2X
3.33	Agonist	0.31	14.57	Number of Aromatic Rings
3.33	Agonist	0.31	16.22	C in CHR2X
3.33	Gs	0.29	12.4	ES-Count: dssC
3.33	Gs	0.34	12.11	H attached to C0sp3 with one X attached to next carbon
3.33	Gs	0.35	13.39	H attached to heteroatom
3.34	Gs	0.28	19.84	ES-Count: sOH
3.34	Gs	0.28	19.79	ES-Count: aasC
3.34	Gs	0.28	12.35	Number of H-Acceptors
3.34	Gs	0.29	15.46	Molecular Surface Area
3.34	Gs	0.29	21.75	O in alcohol
3.34	Gs	0.32	10.74	Number of Atoms
3.34	Gs	0.32	13.32	Molecular Weight
3.34	Gs	0.33	15.17	Number of Rings
3.34	Gs	0.33	13.32	C in CHR3
3.35	Agonist	0.34	22.47	Number of Rotatable Bonds
3.35	Agonist	0.35	23.46	O in phenol, enol, carboxyl OH
3.35	Antagonist	0.21	12.46	Number of H-Donors
3.35	Antagonist	0.24	12.09	H attached to C0sp3 with one X attached to next carbon

Position	Modus	Mutual Information	Test Statistic S	Molecular Descriptor
3.35	Antagonist	0.24	12.6	ES-Count: sOH
3.35	Gi	0.23	15.44	ES-Count: dO
3.35	Gi	0.27	18.37	H attached to heteroatom
3.36	Agonist	0.29	26.94	ES-Count: sssCH
3.36	Agonist	0.29	30.03	Number of Aromatic Bonds
3.36	Agonist	0.3	29.11	Number of Ring Bonds
3.36	Agonist	0.3	14.2	Number of H-Donors
3.36	Agonist	0.31	25.1	H attached to heteroatom
3.36	Agonist	0.31	24.11	Number of Rotatable Bonds
3.36	Agonist	0.32	19.89	Number of Hydrogens
3.36	Agonist	0.33	22.18	H attached to C0sp3 with no X attached next to C
3.36	Agonist	0.33	16.84	C in CHR2X
3.36	Agonist	0.35	24.96	O in alcohol
3.36	Agonist	0.35	27.55	Number of Aromatic Rings
3.36	Agonist	0.36	24.23	Molecular Weight
3.36	Agonist	0.41	32.22	C in CH2R2
3.36	Agonist	0.43	24.52	Number of Atoms
3.36	Agonist	0.45	22.73	O in phenol, enol, carboxyl OH
3.36	Agonist	0.45	22.5	Number of Bonds
3.36	Antagonist	0.2	11.22	Number of H-Acceptors
3.36	Antagonist	0.2	13.28	Number of Hydrogens
3.36	Antagonist	0.2	11.53	Molecular Weight
3.36	Antagonist	0.2	11.87	Number of Ring Bonds
3.36	Antagonist	0.21	9.64	Number of Bonds
3.36	Antagonist	0.21	18.19	Molecular Surface Area
3.36	Antagonist	0.22	11.95	Number of Atoms
3.36	Antagonist	0.23	28.2	H attached to C1sp3 or C0sp2
3.36	Antagonist	0.24	19.08	ES-Count: dssC
3.36	Antagonist	0.24	12.11	Number of Aromatic Bonds
3.36	Antagonist	0.24	20.59	O in phenol, enol, carboxyl OH
3.36	Antagonist	0.25	15	H attached to C0sp3 with no X attached next to C
3.36	Gi	0.26	14.67	ES-Count: sssCH
3.36	Gi	0.27	15.2	Number of Rotatable Bonds
3.36	Gi	0.27	18.09	Number of Hydrogens
3.36	Gi	0.28	16.27	Number of Bonds
3.36	Gi	0.28	18.38	Number of H-Donors
3.36	Gi	0.28	12.86	ES-Count: aaCH
3.36	Gi	0.29	20.15	ES-Count: sOH
3.36	Gi	0.29	19.48	Number of Atoms
3.36	Gi	0.3	18.92	C in CHR2X
3.36	Gi	0.3	17.54	C in R--CH--R
3.36	Gi	0.3	17.63	Number of H-Acceptors
3.36	Gi	0.31	14.27	Molecular Surface Area
3.36	Gi	0.31	25.5	Molecular Weight
3.36	Gi	0.31	20.72	Molecular Polar Surface Area
3.36	Gi	0.31	27.54	Number of Ring Bonds
3.36	Gi	0.31	16.16	ES-Count: ssCH2
3.36	Gi	0.32	21.51	H attached to heteroatom
3.36	Gi	0.32	18.78	H attached to alpha-Cd
3.36	Gi	0.33	22.86	Number of Aromatic Bonds
3.36	Gi	0.33	20.42	C in CH2R2

Position	Modus	Mutual Information	Test Statistic S	Molecular Descriptor
3.36	Gi	0.35	24	H attached to C0sp3 with no X attached next to C
3.36	Gq	0.27	17.5	C in =CHR
3.36	Gq	0.27	16.33	N in Al3N
3.36	Gq	0.27	23.7	ES-Count: sssCH
3.36	Gq	0.28	14.77	C in CH2R2
3.36	Gq	0.33	19.78	ES-Count: dsCH
3.36	Gq	0.35	14.68	C in CH2RX
3.36	Gq	0.37	15.58	Number of Atoms
3.36	Gq	0.37	28.15	Molecular Weight
3.36	Gs	0.3	17.78	Molecular Polar Surface Area
3.36	Gs	0.3	20.71	ES-Count: dsCH
3.36	Gs	0.31	14.66	ES-Count: aasC
3.36	Gs	0.33	14.43	N in Al3N
3.36	Gs	0.36	14.52	ES-Count: aaN
3.36	Gs	0.37	12.71	Number of H-Donors
3.36	Gs	0.39	20.27	N in R--N--R or R--N--X
3.37	Agonist	0.29	24.21	Molecular Polar Surface Area
3.37	Agonist	0.29	29.54	C in R--CR--R
3.37	Agonist	0.29	21.97	Number of Aromatic Rings
3.37	Agonist	0.31	26.1	Number of Ring Bonds
3.37	Agonist	0.32	26.62	H attached to C0sp3 with no X attached next to C
3.37	Agonist	0.33	34.79	C in CH2R2
3.37	Agonist	0.37	31.37	H attached to heteroatom
3.37	Agonist	0.38	24.81	Number of H-Donors
3.37	Agonist	0.39	24.39	ES-Count: dsCH
3.37	Agonist	0.42	21.72	O in phenol, enol, carboxyl OH
3.37	Agonist	0.44	37.02	C in =CHR
3.37	Agonist	0.45	25.68	ES-Count: aasC
3.37	Antagonist	0.21	10.73	Molecular Weight
3.37	Antagonist	0.22	11.62	Number of Ring Bonds
3.37	Antagonist	0.22	11.56	ES-Count: sOH
3.37	Antagonist	0.22	11.83	Number of Bonds
3.37	Antagonist	0.22	9.97	H attached to C0sp3 with no X attached next to C
3.37	Antagonist	0.22	11.09	Number of Rotatable Bonds
3.37	Antagonist	0.23	12.44	C in CH2R2
3.37	Antagonist	0.23	11.47	Molecular Surface Area
3.37	Antagonist	0.23	17.53	Number of H-Acceptors
3.37	Antagonist	0.23	11.14	Number of Atoms
3.37	Antagonist	0.23	12.12	H attached to C1sp3 or C0sp2
3.37	Antagonist	0.25	11.31	ES-Count: dssC
3.37	Antagonist	0.25	14.7	H attached to C0sp3 with one X attached to next carbon
3.37	Antagonist	0.28	14.69	Number of Aromatic Bonds
3.37	Antagonist	0.3	14.08	Number of Rings
3.37	Antagonist	0.3	11.31	Number of H-Donors
3.37	Gi	0.24	26.52	Molecular Surface Area
3.37	Gi	0.24	21.37	H attached to C0sp3 with no X attached next to C
3.37	Gi	0.24	16.27	ES-Count: dO
3.37	Gi	0.25	20.95	Number of Ring Bonds
3.37	Gq	0.28	16.76	C in R--CR--R

Position	Modus	Mutual Information	Test Statistic S	Molecular Descriptor
3.37	Gq	0.31	23	Number of Rings
3.37	Gq	0.35	20.42	C in CHR2X
3.37	Gq	0.35	15.25	ES-Count: aaN
3.37	Gq	0.36	19.02	Number of Bonds
3.37	Gs	0.28	13.52	ES-Count: aasC
3.37	Gs	0.28	13.14	Number of Hydrogens
3.37	Gs	0.29	20.36	Number of Atoms
3.38	Gq	0.31	25.98	Molecular Weight
3.40	Agonist	0.29	24.74	Number of Rings
3.42	Agonist	0.29	23.08	Number of Aromatic Rings
3.42	Agonist	0.29	17.69	O in phenol, enol, carboxyl OH
3.42	Agonist	0.3	33.81	ES-Count: dsCH
3.42	Agonist	0.31	23.04	ES-Count: ssCH2
3.42	Agonist	0.33	24.06	Number of Ring Bonds
3.42	Agonist	0.35	21.87	ES-Count: aasC
3.42	Agonist	0.36	20.6	Molecular Polar Surface Area
3.42	Agonist	0.37	19.99	C in R--CR--R
3.42	Agonist	0.47	42.75	Number of H-Donors
3.42	Antagonist	0.21	13.92	ES-Count: dssC
3.42	Gi	0.24	15.61	ES-Count: sssCH
3.42	Gi	0.24	16.64	H attached to alpha-Cd
3.42	Gi	0.24	16.58	ES-Count: dO
3.42	Gi	0.24	16.17	H attached to C0sp3 with one X attached to next carbon
3.42	Gi	0.24	20.86	C in R-C(=X)-X or X=C=X
3.42	Gi	0.25	17.86	H attached to heteroatom
3.42	Gi	0.25	18.88	ES-Count: dssC
3.42	Gq	0.26	13.02	ES-Count: sssCH
3.42	Gq	0.27	14.38	ES-Count: dsCH
3.42	Gq	0.29	11.85	Molecular Surface Area
3.42	Gq	0.3	24.33	Molecular Weight
3.44	Agonist	0.29	19.18	H attached to C1sp3 or C0sp2
3.44	Agonist	0.33	20.66	Number of Aromatic Rings
3.44	Antagonist	0.2	7.09	Number of Rings
3.44	Antagonist	0.23	13.39	ES-Count: sOH
3.44	Antagonist	0.23	9.51	H attached to heteroatom
3.44	Antagonist	0.25	10.68	ES-Count: sssCH
3.44	Gi	0.24	16.38	C in CHR2X
3.44	Gi	0.28	18.16	H attached to C0sp3 with one X attached to next carbon
3.44	Gq	0.31	14.02	Number of Aromatic Rings
3.44	Gq	0.33	15.01	C in R-C(=X)-X or X=C=X
3.44	Gs	0.3	11.67	H attached to heteroatom
3.44	Gs	0.33	13.28	Number of H-Donors
3.46	Gs	0.29	27.55	H attached to alpha-Cd
3.52	Agonist	0.29	16.01	Molecular Weight
3.52	Antagonist	0.2	6.98	C in CH2R2
3.52	Antagonist	0.22	9.13	ES-Count: sssN
3.55	Gs	0.29	13.33	Number of Rings
3.55	Gs	0.3	10.5	ES-Count: sOH
3.55	Gs	0.31	9.27	H attached to alpha-Cd
3.55	Gs	0.32	13.74	C in =CHR
3.55	Gs	0.32	9.39	H attached to heteroatom
3.55	Gs	0.32	8.52	ES-Count: dsCH

Position	Modus	Mutual Information	Test Statistic S	Molecular Descriptor
4.41	Gq	0.26	9.85	C in R-C(=X)-X or X=C=X
4.41	Gq	0.29	11.39	H attached to C0sp3 with one X attached to next carbon
4.43	Gq	0.26	12.01	Molecular Surface Area
4.43	Gq	0.29	8.97	C in CHR2X
4.43	Gq	0.3	11.94	Molecular Weight
4.46	Agonist	0.29	24.38	Number of Rotatable Bonds
4.46	Agonist	0.29	16.59	O in alcohol
4.46	Antagonist	0.25	27.99	C in CH2R2
4.46	Gi	0.23	16.67	Number of H-Donors
4.46	Gi	0.24	16.41	H attached to C0sp3 with one X attached to next carbon
4.46	Gi	0.24	16.26	Number of Rotatable Bonds
4.46	Gi	0.25	18.95	H attached to heteroatom
4.46	Gi	0.25	18.83	C in CH2R2
4.47	Gs	0.28	13.24	ES-Count: sOH
4.47	Gs	0.28	8.72	C in CH2RX
4.57	Agonist	0.3	20.83	Molecular Weight
4.57	Agonist	0.37	19.04	Molecular Polar Surface Area
4.57	Agonist	0.39	23.43	H attached to heteroatom
4.57	Antagonist	0.21	7.33	Number of Rings
4.57	Antagonist	0.22	9.07	Molecular Surface Area
4.57	Antagonist	0.24	10.57	Number of H-Donors
4.57	Gi	0.25	20.2	C in CHR2X
4.57	Gi	0.29	20.87	ES-Count: sOH
4.57	Gi	0.3	18.8	C in R-C(=X)-X or X=C=X
4.57	Gi	0.31	19.56	C in CH2R2
4.57	Gq	0.28	14.97	Number of Rings
4.57	Gq	0.33	15.69	ES-Count: dO
4.57	Gq	0.33	12.49	C in CHR2X
4.57	Gs	0.29	10.03	C in CHR3
4.57	Gs	0.3	14.76	ES-Count: aasC
4.57	Gs	0.3	14.85	C in CH2RX
4.57	Gs	0.31	15.64	Number of Rings
4.58	Gs	0.28	10.88	Number of H-Acceptors
4.58	Gs	0.28	12.78	ES-Count: aasC
4.58	Gs	0.29	11.01	ES-Count: dssC
4.58	Gs	0.29	10.09	ES-Count: dO
4.58	Gs	0.3	11.31	Molecular Polar Surface Area
4.60	Antagonist	0.21	9.77	Molecular Weight
4.60	Antagonist	0.21	10.37	Number of Ring Bonds
4.60	Antagonist	0.22	11.12	Number of H-Donors
4.60	Antagonist	0.22	10.36	C in CH2R2
4.60	Antagonist	0.22	9.39	H attached to C0sp3 with no X attached next to C
4.60	Antagonist	0.23	9.19	ES-Count: aasC
4.60	Antagonist	0.23	6.85	ES-Count: dssC
4.60	Antagonist	0.23	8.07	ES-Count: ssNH
4.60	Antagonist	0.23	8.15	H attached to heteroatom
4.60	Antagonist	0.25	11.39	ES-Count: dO
4.60	Antagonist	0.26	12.68	H attached to C0sp3 with one X attached to next carbon
4.60	Gi	0.24	16.72	C in R-C(=X)-X or X=C=X

Position	Modus	Mutual Information	Test Statistic S	Molecular Descriptor
4.60	Gi	0.24	16.91	H attached to C0sp3 with one X attached to next carbon
4.60	Gq	0.27	12.02	Number of Atoms
4.60	Gq	0.28	13.94	ES-Count: aasC
4.60	Gq	0.3	8.61	Number of Hydrogens
4.60	Gq	0.3	10.06	C in R-C(=X)-X or X=C=X
5.35	Agonist	0.34	15.56	C in R--CR--R
5.35	Agonist	0.34	16.19	ES-Count: aaN
5.35	Agonist	0.36	11.47	O in phenol, enol, carboxyl OH
5.35	Agonist	0.36	13.62	N in R--N--R or R--N--X
5.35	Agonist	0.37	23.8	Number of Hydrogens
5.35	Agonist	0.37	12.96	C in CH2R2
5.35	Antagonist	0.2	6.17	Number of H-Acceptors
5.35	Antagonist	0.2	7.44	H attached to C1sp3 or C0sp2
5.35	Antagonist	0.2	7.19	H attached to C0sp3 with no X attached next to C
5.35	Antagonist	0.21	7.44	Number of Atoms
5.35	Antagonist	0.21	5.72	Number of Bonds
5.35	Antagonist	0.21	6.1	Number of H-Donors
5.35	Antagonist	0.22	7.75	H attached to C0sp3 with one X attached to next carbon
5.35	Antagonist	0.22	8.57	ES-Count: sssN
5.35	Antagonist	0.23	5.61	N in Al3N
5.35	Antagonist	0.23	4.62	ES-Count: dssC
5.35	Antagonist	0.24	8.58	H attached to heteroatom
5.35	Gi	0.23	10.77	Number of Atoms
5.35	Gi	0.23	9.19	Number of Bonds
5.35	Gi	0.23	7.94	Molecular Surface Area
5.35	Gi	0.24	10.6	Molecular Weight
5.35	Gi	0.24	11.29	H attached to C0sp3 with no X attached next to C
5.35	Gi	0.25	11.23	Number of H-Acceptors
5.35	Gi	0.25	11.18	Number of H-Donors
5.35	Gi	0.25	7.85	Molecular Polar Surface Area
5.35	Gi	0.26	13.47	H attached to alpha-Cd
5.35	Gi	0.27	11.82	ES-Count: sssCH
5.35	Gi	0.28	10.28	ES-Count: aasC
5.35	Gi	0.29	15.47	H attached to heteroatom
5.35	Gi	0.3	15.68	C in CH2R2
5.35	Gi	0.3	11.58	Number of Aromatic Rings
5.35	Gq	0.28	8.19	N in R--N--R or R--N--X
5.35	Gq	0.3	14.42	O in =O
5.35	Gs	0.28	9.5	Number of Rings
5.35	Gs	0.28	11.22	Number of Bonds
5.35	Gs	0.29	10.09	Number of Hydrogens
5.35	Gs	0.29	8.62	Molecular Surface Area
5.35	Gs	0.3	10.08	Number of H-Donors
5.35	Gs	0.32	7.86	H attached to heteroatom
5.36	Antagonist	0.21	2.71	Number of H-Acceptors
5.36	Antagonist	0.21	4.17	ES-Count: sOH
5.36	Gi	0.23	8.92	Molecular Weight
5.36	Gi	0.23	10.57	C in CHR2X
5.36	Gi	0.23	9.11	Number of H-Acceptors
5.36	Gi	0.25	11.99	ES-Count: sssCH

Position	Modus	Mutual Information	Test Statistic S	Molecular Descriptor
5.36	Gi	0.26	8.67	H attached to C0sp3 with no X attached next to C
5.36	Gi	0.27	9.64	ES-Count: aasC
5.36	Gq	0.26	15.03	Number of Bonds
5.36	Gq	0.27	8.22	O in phenol, enol, carboxyl OH
5.36	Gq	0.28	6.46	O in =O
5.36	Gq	0.31	12.08	ES-Count: dO
5.36	Gq	0.31	16.2	Number of Hydrogens
5.36	Gq	0.31	7.84	C in CH2R2
5.36	Gs	0.28	7.66	C in CH2R2
5.36	Gs	0.31	6.62	Molecular Weight
5.36	Gs	0.33	6.84	ES-Count: dssC
5.37	Agonist	0.33	12.5	C in X--CH...X
5.37	Agonist	0.33	14	Number of Rotatable Bonds
5.37	Gq	0.27	14.36	ES-Count: aaN
5.37	Gq	0.33	11.58	H attached to C1sp3 or C0sp2
5.37	Gq	0.33	9.37	Number of Rotatable Bonds
5.37	Gq	0.36	9.24	N in Ar2NH, Ar3N, Ar2N-Al or R...N...R
5.37	Gs	0.28	8.61	Number of Atoms
5.37	Gs	0.29	7.31	Molecular Surface Area
5.37	Gs	0.34	10.27	H attached to alpha-Cd
5.39	Agonist	0.3	16.93	Number of Aromatic Rings
5.39	Agonist	0.31	16.08	H attached to C1sp3 or C0sp2
5.39	Agonist	0.37	25.97	ES-Count: ssCH2
5.39	Antagonist	0.2	6.45	Number of H-Acceptors
5.39	Antagonist	0.2	8.5	H attached to C0sp3 with one X attached to next carbon
5.39	Antagonist	0.21	4.96	ES-Count: dssC
5.39	Antagonist	0.21	8	ES-Count: aasC
5.39	Gi	0.23	15.78	C in CHR2X
5.39	Gi	0.23	15.31	Number of Hydrogens
5.39	Gi	0.23	12.62	Number of Ring Bonds
5.39	Gi	0.23	17.54	Number of Atoms
5.39	Gi	0.24	18.72	H attached to alpha-Cd
5.39	Gi	0.24	16.27	H attached to heteroatom
5.39	Gi	0.24	15.63	Molecular Weight
5.39	Gi	0.24	14.81	C in CH2R2
5.39	Gi	0.25	16.91	Molecular Surface Area
5.39	Gi	0.25	16.61	Molecular Polar Surface Area
5.39	Gi	0.27	16.4	H attached to C0sp3 with one X attached to next carbon
5.39	Gi	0.27	16.51	C in R-C(=X)-X or X=C=X
5.39	Gi	0.27	29.07	N in RCO-N< or >N-X=X
5.39	Gq	0.27	13.71	Number of Bonds
5.39	Gq	0.27	15.76	Number of Ring Bonds
5.39	Gq	0.28	14.22	ES-Count: dO
5.39	Gq	0.29	14.31	Number of Aromatic Rings
5.39	Gq	0.3	12.65	O in =O
5.39	Gq	0.34	12.71	ES-Count: sssCH
5.39	Gq	0.34	10.75	C in CH2R2
5.39	Gq	0.39	12.98	Number of Hydrogens
5.40	Antagonist	0.2	7.54	Number of Atoms
5.40	Antagonist	0.21	9.38	Number of Bonds

Position	Modus	Mutual Information	Test Statistic S	Molecular Descriptor
5.40	Antagonist	0.21	9.81	H attached to C0sp3 with one X attached to next carbon
5.40	Gq	0.28	12.32	Molecular Surface Area
5.40	Gq	0.29	11.6	Number of Bonds
5.40	Gq	0.32	11.14	ES-Count: sssCH
5.40	Gs	0.29	10.72	ES-Count: dssC
5.41	Agonist	0.29	24.23	ES-Count: dsCH
5.41	Agonist	0.29	16.03	ES-Count: ssCH2
5.41	Agonist	0.31	13.43	O in phenol, enol, carboxyl OH
5.41	Agonist	0.34	14.21	Number of Bonds
5.41	Agonist	0.36	22.17	N in Ar2NH, Ar3N, Ar2N-Al or R...N...R
5.41	Antagonist	0.2	8.61	Number of Atoms
5.41	Antagonist	0.2	8.02	ES-Count: dssC
5.41	Antagonist	0.23	6.12	Molecular Surface Area
5.41	Antagonist	0.24	7.93	Molecular Weight
5.41	Antagonist	0.24	7.28	Molecular Polar Surface Area
5.41	Gi	0.26	13.2	H attached to C0sp3 with one X attached to next carbon
5.41	Gi	0.26	17.14	H attached to heteroatom
5.41	Gi	0.28	12.69	Number of H-Acceptors
5.41	Gq	0.26	11.32	Molecular Polar Surface Area
5.41	Gq	0.27	14.45	Number of H-Donors
5.41	Gs	0.3	12.72	H attached to C0sp3 with one X attached to next carbon
5.42	Agonist	0.29	15.27	Number of Atoms
5.42	Agonist	0.3	12.53	Number of H-Donors
5.42	Agonist	0.31	21.14	H attached to heteroatom
5.42	Agonist	0.37	17.77	C in CHR2X
5.42	Agonist	0.42	22.32	Number of Bonds
5.42	Antagonist	0.2	8.12	Number of Atoms
5.42	Antagonist	0.2	7.9	H attached to C1sp3 or C0sp2
5.42	Antagonist	0.21	9.27	Molecular Surface Area
5.42	Antagonist	0.21	9.48	Number of Aromatic Bonds
5.42	Antagonist	0.22	7.87	Number of Ring Bonds
5.42	Antagonist	0.24	8.8	Number of H-Donors
5.42	Antagonist	0.24	7.81	Number of H-Acceptors
5.42	Antagonist	0.25	9.6	ES-Count: dO
5.42	Antagonist	0.25	6.43	C in CH2R2
5.42	Antagonist	0.26	10.34	C in CH2RX
5.42	Antagonist	0.26	8.4	ES-Count: sOH
5.42	Antagonist	0.27	7.88	ES-Count: ssNH
5.42	Gi	0.23	14.99	Molecular Weight
5.42	Gi	0.23	13.14	H attached to heteroatom
5.42	Gi	0.28	15.02	Number of H-Acceptors
5.42	Gi	0.32	16.54	ES-Count: dO
5.42	Gq	0.27	13.46	O in alcohol
5.42	Gq	0.33	13.91	C in R--CR--R
5.42	Gq	0.36	13.43	H attached to C1sp3 or C0sp2
5.42	Gs	0.28	10.16	H attached to alpha-Cd
5.42	Gs	0.29	14.86	Molecular Weight
5.43	Agonist	0.3	15.01	C in R--CR--R
5.43	Agonist	0.34	17.68	ES-Count: dO
5.43	Antagonist	0.21	8.95	Number of Aromatic Bonds

Position	Modus	Mutual Information	Test Statistic S	Molecular Descriptor
5.43	Antagonist	0.22	10.2	ES-Count: ssNH
5.43	Antagonist	0.23	8.55	Number of H-Acceptors
5.43	Antagonist	0.23	13.31	Number of Rings
5.43	Antagonist	0.24	10.6	H attached to heteroatom
5.43	Antagonist	0.26	8.99	C in CH2R2
5.43	Gi	0.24	14.82	Number of Aromatic Bonds
5.43	Gq	0.27	10.04	C in CHR2X
5.43	Gq	0.28	11.22	Molecular Surface Area
5.43	Gs	0.34	11.31	ES-Count: sssCH
5.43	Gs	0.39	12.56	H attached to C0sp3 with no X attached next to C
5.45	Gi	0.23	14.74	Number of H-Acceptors
5.45	Gi	0.26	16.88	Number of Ring Bonds
5.46	Agonist	0.32	17.75	H attached to C0sp3 with no X attached next to C
5.46	Agonist	0.33	23.46	C in CH2R2
5.46	Agonist	0.34	16.77	O in phenol, enol, carboxyl OH
5.46	Agonist	0.35	15.84	ES-Count: ssCH2
5.46	Antagonist	0.2	9.02	ES-Count: dssC
5.46	Antagonist	0.21	10.74	N in Al3N
5.46	Antagonist	0.21	7.05	Number of Bonds
5.46	Antagonist	0.21	6.95	O in =O
5.46	Antagonist	0.22	11.13	Number of H-Donors
5.46	Antagonist	0.23	10.27	ES-Count: ssNH
5.46	Antagonist	0.25	10.9	C in CH2R2
5.46	Antagonist	0.25	7.58	Number of Ring Bonds
5.46	Antagonist	0.28	15.15	H attached to heteroatom
5.46	Gq	0.27	16.8	O in =O
5.46	Gq	0.27	16.75	H attached to C0sp3 with no X attached next to C
5.46	Gq	0.3	14.08	Number of Ring Bonds
5.46	Gq	0.31	11.96	Molecular Weight
5.46	Gq	0.31	13.98	ES-Count: dO
5.46	Gq	0.33	10.59	Number of Atoms
5.46	Gs	0.28	12.01	Number of H-Donors
5.46	Gs	0.29	8.78	Number of Rings
5.46	Gs	0.29	10.44	ES-Count: sOH
5.46	Gs	0.3	12.12	Number of Atoms
5.46	Gs	0.31	12.48	Molecular Weight
5.48	Agonist	0.29	25.87	ES-Count: dsCH
5.48	Gq	0.27	27.66	ES-Count: aaN
5.51	Gs	0.29	12.03	Molecular Polar Surface Area
5.57	Antagonist	0.2	6.96	Number of Aromatic Bonds
5.57	Antagonist	0.21	8.36	ES-Count: dO
5.57	Gq	0.31	13.44	H attached to heteroatom
5.57	Gq	0.32	20.51	ES-Count: aaaC
5.57	Gq	0.33	16.03	N in Ar2NH, Ar3N, Ar2N-Al or R...N...R
5.57	Gs	0.28	12.32	H attached to alpha-Cd
5.57	Gs	0.28	11.2	N in Al3N
5.59	Gq	0.29	10.69	Number of Ring Bonds
5.59	Gq	0.31	10.38	H attached to C0sp3 with one X attached to next carbon
5.60	Gi	0.24	12.7	ES-Count: dO

Position	Modus	Mutual Information	Test Statistic S	Molecular Descriptor
5.60	Gs	0.29	13.61	Molecular Weight
5.60	Gs	0.31	14.13	Number of Rings
5.60	Gs	0.33	10.87	Number of Bonds
5.62	Gs	0.28	15.47	ES-Count: sssCH
5.64	Gs	0.29	8.09	ES-Count: aasC
6.33	Agonist	0.35	21.98	Number of Atoms
6.33	Agonist	0.35	19.96	H attached to heteroatom
6.35	Agonist	0.29	14.97	O in alcohol
6.38	Agonist	0.33	21.23	H attached to alpha-Cd
6.38	Agonist	0.37	24.12	H attached to C1sp3 or C0sp2
6.38	Antagonist	0.22	11.15	Molecular Surface Area
6.38	Antagonist	0.22	11.62	Number of H-Acceptors
6.38	Antagonist	0.23	9.89	Number of Rings
6.38	Antagonist	0.23	11.92	ES-Count: sOH
6.38	Antagonist	0.24	12.08	Number of Bridge Bonds
6.38	Gi	0.25	20.95	Molecular Surface Area
6.38	Gq	0.28	14.51	N in R--N--R or R--N--X
6.38	Gq	0.3	13.88	Number of Ring Bonds
6.38	Gq	0.3	14.34	ES-Count: sssCH
6.39	Gq	0.27	13.51	O in =O
6.39	Gq	0.32	12.73	ES-Count: ssCH2
6.39	Gq	0.32	14.39	ES-Count: sssCH
6.42	Agonist	0.29	18.5	Molecular Weight
6.42	Agonist	0.31	18.55	ES-Count: aaaC
6.42	Antagonist	0.22	11.58	O in =O
6.42	Antagonist	0.23	11.41	ES-Count: dO
6.42	Gi	0.23	19.12	ES-Count: dssC
6.42	Gq	0.26	10.33	ES-Count: dsCH
6.42	Gq	0.27	13.71	ES-Count: ssCH2
6.42	Gq	0.31	14	O in =O
6.45	Gs	0.3	14.93	Molecular Polar Surface Area
6.49	Gi	0.24	13.12	Number of Bridge Bonds
6.49	Gq	0.27	9.79	C in CH2R2
6.49	Gq	0.3	15.17	C in R--CR--R
6.51	Gi	0.23	20.75	Molecular Surface Area
6.52	Agonist	0.3	20.73	C in CH2R2
6.52	Agonist	0.31	31.1	Number of H-Donors
6.52	Agonist	0.31	23.27	Molecular Weight
6.52	Agonist	0.36	32.57	C in CHR2X
6.52	Agonist	0.39	21.72	C in =CHR
6.52	Agonist	0.41	26.99	ES-Count: dsCH
6.52	Antagonist	0.2	8.84	ES-Count: aasC
6.52	Antagonist	0.2	12.5	Number of H-Donors
6.52	Antagonist	0.22	13.52	Number of Rings
6.52	Antagonist	0.25	11.29	O in phenol, enol, carboxyl OH
6.52	Antagonist	0.28	16.72	Molecular Surface Area
6.52	Antagonist	0.28	16.99	Molecular Weight
6.52	Gi	0.23	17.69	Molecular Weight
6.52	Gi	0.24	16.05	H attached to C0sp3 with no X attached next to C
6.52	Gi	0.24	17.45	Number of H-Donors
6.52	Gi	0.24	19.81	C in CHR2X
6.52	Gi	0.25	17.29	C in CH2R2
6.52	Gi	0.25	20.13	H attached to heteroatom

Position	Modus	Mutual Information	Test Statistic S	Molecular Descriptor
6.52	Gi	0.26	21.83	Molecular Surface Area
6.52	Gi	0.31	21.9	ES-Count: ssCH2
6.52	Gi	0.32	20.04	ES-Count: aasC
6.52	Gq	0.26	27.42	H attached to C0sp3 with no X attached next to C
6.52	Gq	0.29	24.44	Number of Atoms
6.52	Gq	0.3	20.16	Number of Bonds
6.52	Gq	0.3	19.6	Molecular Weight
6.52	Gq	0.34	17.54	ES-Count: dsCH
6.52	Gq	0.35	17.28	Number of Rings
6.52	Gs	0.29	18.52	N in R--N--R or R--N--X
6.52	Gs	0.29	9.67	ES-Count: aasC
6.52	Gs	0.3	11.62	Molecular Polar Surface Area
6.52	Gs	0.32	12.23	C in CH2RX
6.52	Gs	0.32	17.65	ES-Count: sssCH
6.52	Gs	0.33	13.75	Number of Rings
6.54	Gq	0.26	11.03	ES-Count: dO
6.55	Agonist	0.29	19.8	Number of Atoms
6.55	Antagonist	0.21	4.85	ES-Count: sOH
6.55	Antagonist	0.21	6.86	ES-Count: dssC
6.55	Antagonist	0.21	6.5	ES-Count: sssCH
6.55	Antagonist	0.21	7.93	ES-Count: dO
6.55	Gq	0.27	8.03	ES-Count: ssCH2
6.55	Gq	0.31	13.44	ES-Count: ssO
6.55	Gs	0.28	7.53	ES-Count: sssCH
6.55	Gs	0.29	7.55	ES-Count: dsCH
6.56	Gq	0.28	14.68	O in phenol, enol, carboxyl OH
6.56	Gq	0.29	13.13	N in Al3N
6.56	Gq	0.33	13.76	C in CH2R2
6.58	Agonist	0.29	16.26	H attached to C0sp3 with one X attached to next carbon
6.58	Agonist	0.33	12.64	C in CHR2X
6.58	Agonist	0.34	11.05	H attached to C1sp3 or C0sp2
6.58	Antagonist	0.2	3.72	H attached to C0sp3 with no X attached next to C
6.58	Antagonist	0.2	5.69	Number of Rings
6.58	Antagonist	0.21	11.3	Number of Bonds
6.58	Antagonist	0.21	4.7	Number of H-Acceptors
6.58	Antagonist	0.21	5.94	Number of H-Donors
6.58	Antagonist	0.21	8.01	C in CH2R2
6.58	Antagonist	0.22	6.19	Number of Atoms
6.58	Antagonist	0.23	6.53	H attached to C1sp3 or C0sp2
6.58	Antagonist	0.23	3.83	ES-Count: sssCH
6.58	Antagonist	0.24	4.98	Molecular Weight
6.58	Antagonist	0.25	5.94	Molecular Surface Area
6.58	Gi	0.23	5.47	C in CH2R2
6.58	Gi	0.24	10.59	H attached to C0sp3 with no X attached next to C
6.58	Gi	0.24	8.18	H attached to alpha-Cd
6.58	Gq	0.27	14.86	Number of Atoms
6.58	Gq	0.28	13.74	Molecular Weight
6.58	Gq	0.31	12.5	H attached to alpha-Cd
6.58	Gq	0.32	11.95	Number of Hydrogens
6.58	Gq	0.38	12.3	Number of Aromatic Rings

Position	Modus	Mutual Information	Test Statistic S	Molecular Descriptor
6.58	Gq	0.4	11.96	H attached to C0sp3 with one X attached to next carbon
6.58	Gs	0.28	7.86	Number of Bonds
6.59	Antagonist	0.21	5.07	Number of H-Acceptors
6.59	Antagonist	0.21	6.33	Number of H-Donors
6.59	Antagonist	0.21	9.13	ES-Count: dssC
6.59	Antagonist	0.21	7.15	C in CH2RX
6.59	Gi	0.23	14.23	C in CHR2X
7.32	Agonist	0.29	10.43	Number of Atoms
7.32	Agonist	0.3	13.37	ES-Count: ssCH2
7.32	Agonist	0.32	9.81	ES-Count: sssCH
7.32	Agonist	0.34	9.47	C in R--CR--R
7.32	Gi	0.23	11.68	Molecular Weight
7.32	Gi	0.24	8.24	ES-Count: sOH
7.32	Gi	0.25	10.52	Molecular Surface Area
7.32	Gi	0.27	10.31	C in CHR2X
7.32	Gq	0.26	7.37	Number of Bonds
7.32	Gq	0.29	14.12	Number of Aromatic Rings
7.32	Gq	0.29	8.8	ES-Count: dssC
7.32	Gq	0.34	9.9	Molecular Weight
7.32	Gq	0.39	11	Number of Rings
7.32	Gq	0.4	9.89	C in R--CR--R
7.32	Gs	0.28	6.78	ES-Count: dsCH
7.32	Gs	0.29	5.64	Molecular Weight
7.32	Gs	0.29	5.92	Number of Atoms
7.33	Antagonist	0.21	6.12	Molecular Surface Area
7.33	Antagonist	0.21	3.12	H attached to C1sp3 or C0sp2
7.33	Antagonist	0.24	5.25	Number of Rings
7.33	Antagonist	0.26	6.28	ES-Count: aasC
7.33	Gq	0.29	10.31	Molecular Surface Area
7.33	Gq	0.3	7.67	ES-Count: dsCH
7.33	Gq	0.32	7.73	C in CH2R2
7.33	Gs	0.29	9.29	Molecular Surface Area
7.33	Gs	0.29	9.44	Number of Hydrogens
7.33	Gs	0.29	9.6	O in phenol, enol, carboxyl OH
7.34	Agonist	0.29	12.73	Number of Atoms
7.34	Agonist	0.32	15.36	H attached to C1sp3 or C0sp2
7.34	Antagonist	0.21	9.85	H attached to heteroatom
7.34	Gs	0.28	9.09	H attached to alpha-Cd
7.34	Gs	0.3	10.63	Number of Bonds
7.34	Gs	0.31	9.75	Molecular Polar Surface Area
7.34	Gs	0.33	12.66	Number of Ring Bonds
7.34	Gs	0.33	7.68	C in CHR3
7.35	Agonist	0.29	18.44	C in R--CR--R
7.35	Antagonist	0.21	4.61	Number of H-Acceptors
7.35	Antagonist	0.25	8.22	H attached to heteroatom
7.35	Antagonist	0.27	10.25	ES-Count: aasC
7.35	Gq	0.26	9.4	ES-Count: dO
7.35	Gs	0.31	6.85	ES-Count: aasC
7.35	Gs	0.31	9.99	C in CHR3
7.35	Gs	0.31	10.15	ES-Count: sssCH
7.35	Gs	0.34	10.51	Molecular Surface Area
7.36	Agonist	0.3	10.34	H attached to heteroatom
7.36	Agonist	0.32	16.71	Number of H-Donors

Position	Modus	Mutual Information	Test Statistic S	Molecular Descriptor
7.36	Agonist	0.33	14.14	Molecular Polar Surface Area
7.36	Agonist	0.37	14.18	C in R--CR--R
7.36	Agonist	0.38	26.68	ES-Count: sssCH
7.36	Antagonist	0.2	7.26	Number of H-Acceptors
7.36	Antagonist	0.21	11.66	H attached to C0sp3 with one X attached to next carbon
7.36	Antagonist	0.22	4.38	ES-Count: aasC
7.36	Antagonist	0.23	7.08	Number of H-Donors
7.36	Antagonist	0.23	5.68	Number of Rings
7.36	Antagonist	0.24	7.01	N in Al3N
7.36	Gi	0.23	9.59	Number of H-Acceptors
7.36	Gi	0.24	11.64	H attached to heteroatom
7.36	Gi	0.24	14.2	H attached to alpha-Cd
7.36	Gi	0.24	6.81	ES-Count: sssCH
7.36	Gi	0.24	14.38	C in CHR2X
7.36	Gi	0.26	13.92	H attached to C0sp3 with no X attached next to C
7.36	Gi	0.27	12.74	C in CH2R2
7.36	Gi	0.31	26.3	Number of Hydrogens
7.36	Gi	0.32	20.14	Number of Bonds
7.36	Gi	0.33	14.84	Number of Atoms
7.36	Gi	0.34	16.17	Molecular Weight
7.36	Gq	0.27	13.16	Number of Bonds
7.36	Gq	0.27	9.44	Molecular Weight
7.36	Gq	0.3	10.41	Number of Hydrogens
7.36	Gq	0.32	9.21	H attached to alpha-Cd
7.36	Gq	0.35	9.88	H attached to C1sp3 or C0sp2
7.36	Gs	0.28	10.49	N in R--N--R or R--N--X
7.38	Agonist	0.4	20.41	Molecular Weight
7.38	Agonist	0.4	32.44	Number of Rotatable Bonds
7.38	Agonist	0.42	23.82	ES-Count: ssO
7.38	Antagonist	0.21	10.17	Molecular Surface Area
7.38	Antagonist	0.22	8.46	C in CH2R2
7.38	Antagonist	0.25	12.86	Number of Rings
7.38	Antagonist	0.27	13.14	Number of Aromatic Bonds
7.38	Gi	0.28	18.92	Number of Bridge Bonds
7.38	Gi	0.34	23.06	Molecular Surface Area
7.38	Gq	0.26	12.89	ES-Count: aaaC
7.38	Gq	0.28	21.46	Number of Rotatable Bonds
7.38	Gq	0.34	14.3	ES-Count: sssN
7.38	Gq	0.36	16.94	C in CH2RX
7.38	Gs	0.3	9.71	H attached to C1sp3 or C0sp2
7.39	Antagonist	0.2	9.39	H attached to C0sp3 with no X attached next to C
7.39	Antagonist	0.21	11.32	ES-Count: dO
7.39	Antagonist	0.22	7.26	Number of Aromatic Bonds
7.39	Gq	0.26	10.69	Number of Ring Assemblies
7.39	Gq	0.27	15.56	N in RCO-N< or >N-X=X
7.40	Agonist	0.29	34.93	ES-Count: dsCH
7.40	Agonist	0.3	17.99	C in CHR2X
7.40	Agonist	0.3	23.02	Molecular Weight
7.40	Agonist	0.32	24.67	H attached to heteroatom
7.40	Agonist	0.36	17.19	Number of H-Donors
7.40	Agonist	0.4	31.21	Number of Ring Bonds

Position	Modus	Mutual Information	Test Statistic S	Molecular Descriptor
7.40	Antagonist	0.21	8.75	N in Al3N
7.40	Antagonist	0.21	14.77	Number of Ring Bonds
7.40	Gq	0.26	16.37	Molecular Weight
7.40	Gq	0.29	28.61	Number of Bonds
7.40	Gq	0.31	18.39	Number of Aromatic Rings
7.40	Gq	0.37	27.26	Molecular Surface Area
7.40	Gs	0.28	10.96	Molecular Weight
7.40	Gs	0.3	10.02	C in CHR3
7.42	Agonist	0.29	17.69	Number of H-Donors
7.42	Agonist	0.31	27.51	Number of Hydrogens
7.42	Agonist	0.33	22.55	N in R--N--R or R--N--X
7.42	Agonist	0.36	21.1	ES-Count: aaN
7.42	Agonist	0.42	22.59	C in CHR2X
7.42	Antagonist	0.21	9.59	O in =O
7.42	Antagonist	0.23	13.3	Number of H-Acceptors
7.42	Antagonist	0.23	11.61	Number of Ring Bonds
7.42	Antagonist	0.24	13.14	Number of H-Donors
7.42	Antagonist	0.26	12.69	Number of Atoms
7.42	Gi	0.25	22.55	H attached to C0sp3 with no X attached next to C
7.42	Gq	0.28	13.98	ES-Count: dssC
7.42	Gq	0.29	17.77	Number of Ring Bonds
7.42	Gq	0.29	15.04	Molecular Surface Area
7.42	Gs	0.28	16.86	Number of Hydrogens
7.42	Gs	0.29	9.55	ES-Count: dsCH
7.42	Gs	0.3	10.07	H attached to heteroatom
7.42	Gs	0.3	13.66	C in =CHR
7.43	Agonist	0.3	19.92	Number of H-Donors
7.43	Agonist	0.31	41.15	C in =CHR
7.43	Antagonist	0.2	14.49	N in Al3N
7.43	Antagonist	0.21	14.98	ES-Count: sssCH
7.43	Gs	0.28	11.74	Molecular Polar Surface Area
7.44	Gs	0.28	8.49	Number of H-Donors
7.44	Gs	0.31	9.22	H attached to C0sp3 with one X attached to next carbon
7.47	Agonist	0.3	12.15	C in CH2R2
7.47	Agonist	0.3	25.99	Molecular Polar Surface Area
7.47	Agonist	0.3	25.4	Number of H-Donors
7.47	Agonist	0.3	17.56	H attached to heteroatom
7.47	Agonist	0.34	23.07	Number of Ring Bonds
7.47	Agonist	0.35	20.26	O in phenol, enol, carboxyl OH
7.47	Agonist	0.36	23.44	C in CHR3
7.47	Agonist	0.38	33.97	ES-Count: aasC
7.47	Gi	0.23	11.85	Number of Atoms
7.47	Gi	0.24	13.83	H attached to heteroatom
7.47	Gi	0.25	14.71	C in CHR2X
7.47	Gi	0.25	13.21	Molecular Surface Area
7.47	Gi	0.26	18.88	Molecular Weight
7.47	Gq	0.27	14.05	ES-Count: aasC
7.47	Gq	0.29	13.47	ES-Count: sssCH
7.54	Agonist	0.29	26.37	H attached to C0sp3 with no X attached next to C
7.54	Agonist	0.31	24.44	Number of Aromatic Rings
7.54	Agonist	0.31	24.5	ES-Count: ssCH2

Position	Modus	Mutual Information	Test Statistic S	Molecular Descriptor
7.54	Agonist	0.34	20.21	ES-Count: dsCH
7.54	Agonist	0.37	21.41	Number of H-Donors
7.54	Agonist	0.37	21.49	C in =CHR
7.54	Agonist	0.4	29.08	ES-Count: aasC
7.54	Antagonist	0.2	16.94	H attached to C0sp3 with no X attached next to C
7.54	Antagonist	0.2	10.48	Number of Aromatic Rings
7.54	Antagonist	0.21	13.96	Number of Hydrogens
7.54	Antagonist	0.21	9.13	ES-Count: dO
7.54	Antagonist	0.22	11.94	ES-Count: dssC
7.54	Antagonist	0.22	9.37	Number of Bonds
7.54	Antagonist	0.23	11.55	ES-Count: aasC
7.54	Antagonist	0.25	14.75	H attached to C1sp3 or C0sp2
7.54	Gi	0.23	16.39	Number of H-Donors
7.54	Gi	0.24	16.88	Number of Bonds
7.54	Gi	0.24	19.42	Number of Hydrogens
7.54	Gi	0.24	18.06	ES-Count: aasC
7.54	Gi	0.24	13.93	C in R-C(=X)-X or X=C=X
7.54	Gi	0.24	18.46	Number of Ring Bonds
7.54	Gi	0.25	27.04	H attached to C0sp3 with no X attached next to C
7.54	Gi	0.26	18.53	Molecular Surface Area
7.54	Gi	0.28	19.28	C in CHR2X
7.54	Gi	0.29	22.34	ES-Count: ssCH2
7.54	Gi	0.3	18.83	H attached to heteroatom
7.54	Gi	0.31	18.72	Molecular Weight
7.54	Gq	0.26	19.65	ES-Count: dssC
7.54	Gq	0.3	13.08	Number of Bonds
7.54	Gq	0.31	22.38	Number of H-Donors
7.54	Gq	0.34	14.92	ES-Count: dO
7.54	Gq	0.39	16.59	Number of Hydrogens
7.54	Gq	0.41	14.9	C in CH2R2
7.54	Gs	0.28	11.76	H attached to C0sp3 with one X attached to next carbon
7.54	Gs	0.28	15.52	Number of Aromatic Rings
7.54	Gs	0.3	13.04	Number of Atoms
7.54	Gs	0.3	12.93	Molecular Weight
7.54	Gs	0.32	12.34	O in phenol, enol, carboxyl OH
7.54	Gs	0.32	11.52	ES-Count: sssCH
7.56	Agonist	0.29	17.84	Molecular Surface Area
7.56	Agonist	0.3	24.02	O in alcohol
7.56	Agonist	0.3	17.91	C in CH2R2
7.56	Agonist	0.32	18.73	ES-Count: ssCH2
7.56	Antagonist	0.2	11.12	H attached to C0sp3 with no X attached next to C
7.56	Antagonist	0.23	11.39	Number of H-Acceptors
7.56	Antagonist	0.23	11.98	ES-Count: sOH
7.56	Antagonist	0.24	14.58	C in CH2R2
7.56	Gi	0.23	19.58	C in CHR2X
7.56	Gi	0.23	16.11	H attached to heteroatom
7.56	Gi	0.23	17.8	O in =O
7.56	Gq	0.29	18.28	Molecular Weight
7.56	Gq	0.32	16.37	ES-Count: ssCH2
7.56	Gq	0.33	17.01	C in CH2R2

Position	Modus	Mutual Information	Test Statistic S	Molecular Descriptor
8.47	Agonist	0.29	16.6	H attached to C0sp3 with no X attached next to C
8.47	Agonist	0.3	23.37	C in =CHR
8.47	Agonist	0.3	22.96	ES-Count: dsCH
8.47	Agonist	0.3	29.62	C in CH2R2
8.47	Agonist	0.31	17.54	H attached to heteroatom
8.47	Agonist	0.33	38.83	Molecular Polar Surface Area
8.47	Agonist	0.33	34.75	Number of H-Donors
8.47	Agonist	0.34	21.17	ES-Count: ssCH2
8.47	Agonist	0.37	28.83	C in CHR2X
8.47	Agonist	0.46	38.22	Number of Aromatic Rings
8.47	Antagonist	0.21	11.46	H attached to heteroatom
8.47	Antagonist	0.22	13.43	Number of Ring Bonds
8.47	Gq	0.26	11.05	Number of Hydrogens
8.49	Gq	0.27	10.03	Molecular Surface Area
8.49	Gs	0.28	10.45	ES-Count: sssCH
8.49	Gs	0.29	10.47	Molecular Polar Surface Area
8.50	Gs	0.28	23.18	ES-Count: sssCH
8.50	Gs	0.3	23.58	Number of H-Acceptors
8.53	Agonist	0.34	16.31	O in alcohol
8.53	Agonist	0.34	17.4	Number of H-Donors
8.53	Antagonist	0.2	5.96	H attached to heteroatom
8.53	Antagonist	0.21	9.11	ES-Count: aasC
8.53	Antagonist	0.21	11.81	H attached to C0sp3 with one X attached to next carbon
8.53	Antagonist	0.23	11.26	H attached to alpha-Cd
8.53	Gi	0.23	15.11	C in R--CH--R
8.53	Gi	0.23	23.1	Number of Rotatable Bonds
8.53	Gi	0.24	16.69	Number of Hydrogens
8.53	Gi	0.24	18.08	ES-Count: aaCH
8.53	Gi	0.25	18.35	H attached to alpha-Cd
8.53	Gi	0.25	21.28	Number of Atoms
8.53	Gi	0.25	11.27	Number of Bonds
8.53	Gi	0.25	18.87	Number of H-Acceptors
8.53	Gi	0.25	12.84	ES-Count: sssCH
8.53	Gi	0.25	17.23	Molecular Weight
8.53	Gi	0.25	19.97	C in CHR2X
8.53	Gi	0.26	15.94	Molecular Surface Area
8.53	Gi	0.26	16.17	C in CH2R2
8.53	Gi	0.26	16.23	O in =O
8.53	Gi	0.27	14.62	ES-Count: ssCH2
8.53	Gi	0.27	15.34	C in R-C(=X)-X or X=C=X
8.53	Gi	0.28	16.82	ES-Count: dO
8.53	Gi	0.28	15.52	Number of H-Donors
8.53	Gi	0.28	17.85	Molecular Polar Surface Area
8.53	Gi	0.29	17.24	ES-Count: sOH
8.53	Gi	0.31	19.58	ES-Count: dssC
8.53	Gq	0.27	15.33	C in CH2R2
8.53	Gq	0.28	10.95	Molecular Polar Surface Area
8.53	Gq	0.37	11.01	Number of Aromatic Rings
8.53	Gq	0.37	13	H attached to alpha-Cd
8.55	Gq	0.26	7.73	O in alcohol
8.55	Gq	0.26	7.93	Number of Hydrogens
8.56	Antagonist	0.2	6.39	Number of Bridge Bonds

Position	Modus	Mutual Information	Test Statistic S	Molecular Descriptor
8.56	Gq	0.27	10.04	N in R--N--R or R--N--X
8.56	Gq	0.3	11.98	C in CH2R2
Lp2.48	Gs	0.29	10.94	H attached to C0sp3 with one X attached to next carbon
Lp2.48	Gs	0.29	8.98	C in R--CX--X
Lp2.48	Gs	0.3	9.44	Number of Aromatic Rings
Lp2.48	Agonist	0.29	21.81	Number of Rings
Lp2.48	Gs	0.3	11.5	H attached to alpha-Cd
Lp2.49	Agonist	0.29	17.16	C in CHR3
Lp2.49	Agonist	0.31	10.25	O in phenol, enol, carboxyl OH
Lp2.49	Agonist	0.31	11.93	ES-Count: sssCH
Lp2.49	Agonist	0.31	16.26	H attached to heteroatom
Lp2.49	Agonist	0.33	13.11	Molecular Polar Surface Area
Lp2.49	Agonist	0.33	21.87	Number of Rings
Lp2.49	Agonist	0.28	13.23	Number of Aromatic Rings
Lp2.49	Gq	0.26	6.35	ES-Count: sssCH
Lp2.49	Gq	0.26	14.52	Molecular Polar Surface Area
Lp2.49	Gq	0.27	8.56	ES-Count: aasC
Lp2.49	Gq	0.3	9.42	Number of Atoms
Lp2.49	Gq	0.32	9.89	Molecular Surface Area
Lp2.49	Gq	0.33	18.84	Number of Aromatic Rings
Lp2.49	Gq	0.34	9.52	C in CHR3
Lp2.49	Gs	0.29	16.4	C in =CHR
Lp2.49	Gs	0.29	11.05	Number of Aromatic Rings
Lp2.49	Gs	0.3	17.16	Number of Hydrogens
Lp2.49	Gs	0.32	10.84	Molecular Polar Surface Area
Lp2.49	Gs	0.33	11.9	Molecular Surface Area
Lp2.49	Gs	0.35	10.79	ES-Count: sOH
Lp2.49	Gs	0.35	11.55	O in alcohol
Lp2.49	Gs	0.37	13.42	Number of Atoms
Lp2.49	Gs	0.38	9.57	C in CHR3
Lp2.49	Gs	0.39	12.41	Molecular Weight
Lp2.49	Gs	0.39	11.87	ES-Count: aasC
Lp2.51	Agonist	0.32	15.13	O in phenol, enol, carboxyl OH
Lp2.51	Agonist	0.35	24.63	Number of H-Acceptors
Lp2.51	Agonist	0.42	23.3	O in alcohol
Lp2.51	Agonist	0.43	17.87	C in CHR3
Lp2.51	Agonist	0.43	17.52	H attached to heteroatom
Lp2.51	Agonist	0.31	23.7	ES-Count: ssCH2
Lp2.51	Gi	0.27	12.58	Number of Atoms
Lp2.51	Gi	0.27	9.56	C in CHR2X
Lp2.51	Gi	0.27	8.9	Molecular Polar Surface Area
Lp2.51	Gq	0.3	12.31	Number of Atoms
Lp2.51	Gq	0.32	10.41	Molecular Surface Area
Lp2.51	Gq	0.35	11.47	ES-Count: aaN
Lp2.51	Gs	0.28	13.18	C in CH2R2
Lp2.51	Gs	0.29	8.92	ES-Count: ssCH2
Lp2.51	Gs	0.29	13.34	Number of Ring Bonds
Lp2.51	Gs	0.29	10.62	ES-Count: aaCH
Lp2.51	Gs	0.29	10.9	Molecular Surface Area
Lp2.51	Gs	0.29	10.2	Number of Hydrogens
Lp2.51	Gs	0.29	14.23	ES-Count: dsCH
Lp2.51	Gs	0.3	12.09	Number of Atoms
Lp2.51	Gs	0.3	12.15	Number of Bonds

Position	Modus	Mutual Information	Test Statistic S	Molecular Descriptor
Lp2.51	Gs	0.35	8.72	Molecular Weight
Lp2.51	Gs	0.36	11.04	ES-Count: sssCH
Lp2.51	Gs	0.36	8.99	Number of H-Acceptors
Lp2.51	Gs	0.36	10.17	C in =CHR
Lp2.51	Gs	0.37	7.54	Number of Rings
Lp2.51	Gs	0.37	9.55	O in alcohol
Lp2.51	Gs	0.37	11.12	H attached to C0sp3 with one X attached to next carbon
Lp2.51	Gs	0.38	12.18	ES-Count: sOH
Lp2.52	Antagonist	0.2	25.12	ES-Count: sOH
Lp2.52	Antagonist	0.2	9.96	ES-Count: dssC
